# Automated COVID-19 detection with convolutional neural networks

**DOI:** 10.1038/s41598-023-37743-4

**Published:** 2023-06-30

**Authors:** Aphelele Dumakude, Absalom E. Ezugwu

**Affiliations:** 1 School of Mathematics, Statistics, and Computer Science, University of KwaZulu-Natal, King Edward Avenue, Pietermaritzburg Campus, Pietermaritzburg, 3201 KwaZulu-Natal South Africa; 2 Unit for Data Science and Computing, North-West University, 11 Hoffman Street, Potchefstroom, 2520 South Africa

**Keywords:** Infectious diseases, Computer science, Information technology, Scientific data

## Abstract

This paper focuses on addressing the urgent need for efficient and accurate automated screening tools for COVID-19 detection. Inspired by existing research efforts, we propose two framework models to tackle this challenge. The first model combines a conventional CNN architecture as a feature extractor with XGBoost as the classifier. The second model utilizes a classical CNN architecture with a Feedforward Neural Network for classification. The key distinction between the two models lies in their classification layers. Bayesian optimization techniques are employed to optimize the hyperparameters of both models, enabling a “cheat-start” to the training process with optimal configurations. To mitigate overfitting, transfer learning techniques such as Dropout and Batch normalization are incorporated. The CovidxCT-2A dataset is used for training, validation, and testing purposes. To establish a benchmark, we compare the performance of our models with state-of-the-art methods reported in the literature. Evaluation metrics including Precision, Recall, Specificity, Accuracy, and F1-score are employed to assess the efficacy of the models. The hybrid model demonstrates impressive results, achieving high precision (98.43%), recall (98.41%), specificity (99.26%), accuracy (99.04%), and F1-score (98.42%). The standalone CNN model exhibits slightly lower but still commendable performance, with precision (98.25%), recall (98.44%), specificity (99.27%), accuracy (98.97%), and F1-score (98.34%). Importantly, both models outperform five other state-of-the-art models in terms of classification accuracy, as demonstrated by the results of this study.

## Introduction

Given the limited availability of adequate equipment in hospitals, it is crucial to have a quick and efficient diagnosis method to prevent the spread of COVID-19, improve medical therapy effectiveness, and enhance chances of survival without intensive care. Currently, the primary screening tool for COVID-19 is the polymerase chain reaction and reverse transcriptase (RT-PCR) method, which detects the presence of SARS CoV-2 ribonucleic acid (RNA) in respiratory tract samples^1^. However, many countries face challenges in providing sufficient testing capacity, leading to delays in obtaining accurate results, with testing primarily reserved for individuals with obvious symptoms. To address these limitations, there is a need for faster and more reliable screening methods, such as imaging-based approaches, that can complement or replace PCR testing. These screening techniques can be used alongside PCR testing to enhance diagnostic certainty or serve as an alternative in areas where PCR testing is not easily accessible. In response to the outbreak, researchers have expedited the development of COVID-19 detection models utilizing artificial intelligence to assist clinicians^2^. These models aim to accurately detect COVID-19 in patients and deliver test results in the shortest possible time.

Deep learning workflows have significantly advanced in recent years, starting with the introduction of the AlexNet convolutional neural network in 2012^3^. Convolutional neural networks (CNNs) offer distinct advantages in image processing as they can extract features independently, eliminating the need for feature descriptors or specific extraction methods. Unlike traditional machine learning techniques, CNNs require minimal image pre-processing and can automatically learn optimal data representations directly from raw images. This characteristic makes CNNs a more unbiased and objective approach. CNNs have demonstrated remarkable performance across various domains, including medical analysis using images from MRI, microscopy, CT scans, ultrasound, X-rays, and mammography^4–10^. CNNs have been effectively applied to solve segmentation and classification problems, as well as image synthesis tasks^11–14^. Notably, significant progress has been made in lung analysis using deep learning architectures in studies^15,16^. These studies share similarities with COVID-19 research, as both involve extracting crucial information from lung images, with a specific interest in detecting trace ground glass opacity indicative of COVID-19^17^. However, classifying CT lung imaging between COVID-19 and non-COVID-19 patients can pose substantial challenges, particularly when pneumonia-related lung damage from various causes coexists.

The challenges brought about by COVID-19, as mentioned earlier, and the urgent need for fast and accurate testing methods have sparked increased research on deep learning. The success of deep learning in various fields, as demonstrated by previous studies^18,19^, has motivated us to delve deep into deep learning and seek a solution that can aid clinicians in the battle against COVID-19. Specifically, we propose a comprehensive investigation into the classification of COVID-19 from chest CT images, which fall into three classes: COVID-19-infected patients, pneumonia-acquired patients, and normal patients without any lung disease. The goal of this study is to develop a precise classification model that can detect COVID-19 from CT images, a widely explored medical imaging technique that allows for non-invasive visualization of the object's interior^20^.

To elaborate on our study objectives, we employed a transfer learning strategy in the development of our COVID-19 detection model. Transfer learning has been extensively used in the literature with remarkable success, and it is worth exploring due to its potential to enhance our model’s performance, as mentioned in^21^. This approach allowed us to utilize the weights of another pre-trained model, which was trained on a large dataset like ImageNet-21 k^16^. By leveraging transfer learning, we could initialize our model with optimized weights that had already been trained on large datasets, enabling it to recognize general/basic features and be customized for the specific application at hand. This not only saved us training time but also eliminated the need to relearn general features and perform calculations on convolving the image by the kernel and activation functions^22,23^. The work presented in this study contributes to the advancement of the medical field by developing an automated COVID-19 detection model that can aid in testing and controlling the spread of the virus. Our aim was to develop both a traditional Convolutional Neural Network (CNN) and a hybrid CNN-XGBoost model for the classification of COVID-19. Subsequently, we compared our proposed models with those from the study in^24^. The technical contributions of this study can be summarized as follows:

- Performing hyperparameter optimization using Bayesian optimization technique for CNN and XGBoost.
- Developing a hybrid CNN-XGBoost-based transfer learning model that achieves high classification accuracy.
- Developing a stand-alone CNN-based transfer learning model that achieves high classification accuracy.
- Conducting a comparative analysis between our two proposed models, the hybrid CNN-XGBoost and the stand-alone CNN model.
- Performing a comparative analysis of the proposed models with existing state-of-the-art models from the literature.

The remaining sections of this paper are organized as follows: Sect.  “Related works” presents a review of related studies, with a focus on the current gaps in the literature. The methodology of our proposed CNN model is described in Sect.  “Methodology”. In Sect.  “Experimentation and evaluation metrics”, we discuss the computational resources used and the dataset applied in our experimentation. The results obtained from the conducted experiment are analyzed comparatively in Sect.  “Results and discussion”. Finally, in Sect.  “Conclusion”, we present the conclusion of the study, highlighting potential avenues for future research.

## Related works

This section of the work commences with an extensive review of the COVID-19 literature, specifically focusing on deep neural networks (DNNs) and the techniques employed to enhance their performance in COVID-19 classification tasks. These techniques include transfer learning, image pre-processing, data augmentation, and model generalization. By conducting a thorough literature survey, we gather valuable insights into how DNNs and machine learning algorithms have been utilized for the detection and classification of COVID-19 in lung images. The primary objective of this literature review is twofold: to understand the advancements made by previous studies in the field and to identify existing gaps that we can address and contribute to.

Through this literature review, we aim to build a solid foundation of knowledge by examining the accomplishments of other researchers in the domain. Additionally, we seek to identify areas where further research is needed, thus enabling us to make meaningful contributions and fill the existing gaps in the literature.

### Image pre-processing

In a recent research paper^25^, an initial comparative investigation of various standard CNN models was conducted to identify an appropriate model for detecting COVID-19. The authors selected the VGG-19 model and optimized it for the imaging modalities to address the challenges posed by the rare and complex COVID-19 datasets. The study emphasized the difficulties in utilizing the latest COVID-19 datasets due to their size and image quality, which adversely affected the trainability of complex models. To overcome these challenges, the authors proposed an image pre-processing step that aimed to remove noise from the images and create a reliable dataset for deep learning models. The reported results indicated that ultrasound images achieved higher detection accuracy compared to X-ray and CT scans.

The findings from the study^25^ revealed that deeper networks struggled to train effectively when the data was limited, leading to reduced consistency across the three imaging modes. The study observed precision levels of up to 86% for X-rays, 100% for ultrasound, and 84% for CT scans. The proposed model demonstrated superior performance in detecting COVID-19 compared to pneumonia and normal cases. However, the authors noted that the work could be further enhanced by performing lung segmentation on all image samples to reduce noise and sampling bias, particularly for ultrasound images. The CT scans, which were obtained from inconsistent patient locations, yielded lower results compared to X-ray and ultrasound.

Furthermore, the authors^25^ presented a classification of the three widely used imaging modes (X-ray, ultrasound, and CT scan) to illustrate the application of transfer learning in the COVID-19 detection system. This work aims to support practitioners and researchers in developing deep learning models that assist healthcare professionals in making informed treatment decisions. The study also introduces a pre-processing image pipeline for deep learning-based prediction models, which enhances the quality of images in the dataset. Additionally, the suitability of typical deep learning models for the limited dataset was investigated to select a model for the proposed image classification demonstration across various imaging modes.

In another study^26^, two deep-learning architectures were proposed for automatically detecting COVID-19-positive patients from chest X-ray (CXR) images. The authors employed a pre-processing technique called lung segmentation to improve the quality of CT scans, and the enhanced images were utilized in the two proposed models. Both architectures utilized the AlexNet architecture and employed transfer learning, with an artificial neural network (ANN) used for automatic chest image pre-processing. The second architecture featured a hybrid structure incorporating a Bidirectional Long Short-Term Memory (BiLSTM) layer, which considered temporal features. This second hybrid architecture achieved a COVID-19 classification accuracy of 98.70%, surpassing the accuracy of the first design, which was 98.14%. The study concluded that automatic lung segmentation using ANNs can generate robust features for building a CNN-based transfer learning-BiLSTM network, enabling early diagnosis of COVID-19 infection.

Artificial intelligence relies on image segmentation to ensure accurate predictions by the model, as noise or unrelated patterns in the image can lead to inaccuracies. When evaluating the presence of COVID-19 in CXR images, it is more effective to focus solely on the lung region. However, since the lungs are not located in the same area in raw images, each image must be segmented separately to isolate the lung region. Manual segmentation is not feasible due to the limited size of the COVID-19 radiography database, which contains only 2905 images. Therefore, automatic segmentation methods are preferred. To augment the dataset and improve classification accuracy, the authors of the study^26^ adopted an image rotation technique and employed ANN pre-processing methods. While these techniques offer more diverse data and enhance classification accuracy, they also come with drawbacks such as increased memory requirements, costly conversion calculations, and longer training times^27,28^.

The authors noted that many studies neglect lung segmentation and feed the network with unprocessed data. Consequently, the retrieved features from CXR images become complicated for the model to classify accurately. In contrast, the proposed solution in^26^ achieved excellent classification accuracy and faster convergence by incorporating automatic lung segmentation and a hybrid architecture design. The study^29^ further confirmed that BiLST (Bidirectional Long Short-Term Memory) significantly influenced classification accuracy, more so than the convolutional layer. This finding aligns with^26^, where only BiLSTM was used for classification while CNN was utilized for feature extraction. Compared to other studies, this hybrid architecture demonstrated high classification success^26^. Although the hybrid architecture outperformed the non-hybrid architecture with accuracies of 98.70% and 98.14% respectively, the scarcity of datasets limited the study’s findings. Deep neural network architectures heavily rely on large datasets to achieve reliable and satisfactory results, as they are data-hungry by nature.

In^30^, a novel DNN architecture with a human–machine collaborative design approach was proposed for COVID-19 detection from CXR images. The COVID-Net architecture employed a lightweight projection-expansion-projection-extension (PEPX) design, which increased representational capacity while reducing computational complexity. COVID-Net was one of the first open-source network designs developed for COVID-19 detection, promoting reproducibility. The study also introduced an explainability approach to gain insights into the critical elements associated with COVID-19 cases and assist clinicians in the screening process. COVID-Net was transparently validated to ensure that decisions were based on relevant data from the CXR images. The COVIDx dataset was used to train the DNN architectures, and before training, the chest CXR images underwent enhancement by removing embedded textual information. Data augmentation techniques such as horizontal flip, zoom, and intensity shift were employed to introduce additional variations in the dataset. According to the published findings^30^, COVID-Net exhibited significantly lower computational and architectural complexity compared to VGG-19 and ResNet-50 architectures. Specifically, COVID-Net required approximately 2.37 times fewer MAC (Multiply-Accumulate) operations than ResNet-50 and only around 12 times as many as VGG-19. The results demonstrated the advantages of using lightweight PEPX design patterns in COVID-Net over other designs like VGG-19 and ResNet-50, including other types of lightweight design patterns such as bottleneck patterns. COVID-Net achieved considerably higher test accuracy and COVID-19 sensitivity compared to VGG-19 and ResNet-50. The sensitivity of COVID-Net was more than 32% higher than that of VGG-19 and 8% higher than that of ResNet-50. The test results clearly highlight the benefits of COVID-selective Net’s extensive long-range connections and diverse architecture. These architectural choices offer a robust representational capacity specifically tailored for the task at hand, while also streamlining the model's training process. The results serve as evidence for the advantages derived from the distinctive design decisions made during the machine-driven design exploration phase of the human–machine collaborative design technique employed to build COVID-Net.

Furthermore, the results demonstrate that COVID-Net has achieved a high sensitivity score of 91.0% for detecting COVID-19 cases. This is crucial in order to minimize the number of missed COVID-19 cases during the development of deep neural network architectures. Additionally, COVID-high Net has shown a low prevalence of false positive COVID-19 detections, with a positive predictive value (PPV) of 98.9% for COVID-19 instances. This high PPV is important to prevent an excess of false positives that would burden the healthcare system with additional PCR testing and treatment. Based on these findings, the proposed COVID-Net model performs well in detecting COVID-19 cases in chest X-ray (CXR) images. The authors suggest that incorporating additional data could further improve the performance and generalization of the model across all image classes.

Another study, referenced as^20^, explores image pre-processing techniques in the context of computer-aided diagnosis (CAD) using CXR images. The authors of^20^ developed and evaluated a method to differentiate between patients infected with pneumonia and those affected by COVID-19. As CXR images often contain irrelevant diaphragm regions for COVID-19 detection, the authors proposed two image pre-processing techniques: bilateral low-pass filtering and histogram equalization algorithm, to remove the diaphragm. The filtered images were then fed into a convolutional neural network (CNN) model based on transfer learning, consisting of three input channels, to classify the CXR images into three categories: COVID-19 pneumonia, community-acquired pneumonia without COVID-19, and normal (non-pneumonia) cases.

The dataset used in this study contained an equal number of samples for each class, which were randomly divided into training, validation, and testing sets. The presented CAD scheme based on CNN achieved an overall classification accuracy of 94.5% for the three mentioned classes, with a 95% confidence interval of [0.93, 0.96]. When classifying samples with and without COVID-19 infection, the CAD scheme achieved a sensitivity of 98.4% and a specificity of 98.0%. However, the authors noted that without the two image pre-processing steps, the classification accuracy dropped to 88.0%. To enhance the accuracy of diagnosing COVID-19 pneumonia, this study demonstrated the development of a deep-learning CAD scheme for CXR images, incorporating image pre-processing steps and dataset augmentation.

Furthermore, the study^20^ employed transfer learning, using a pre-trained VGG-16 model that had been trained on a large dataset of 14 million images from the ImageNet dataset. This approach helps mitigate overfitting and underfitting issues when working with a small training dataset. The utilization of established CNN models trained on diverse datasets is considered a preferable strategy in such cases. While the study yielded promising results, several limitations were identified:

- The performance and robustness of the proposed CAD scheme should be further validated using additional large and diverse datasets, considering the variations in COVID-19 cases.
- The image pre-processing techniques explored in this study may not be optimal or the most efficient. Future research may need to evaluate and compare various image pre-processing techniques.
- Investigating image pre-processing and segmentation techniques to extract the region of interest (lung area) is necessary. Some images contain unnecessary information, such as the diaphragm, which should be removed to enable the model to focus on the relevant areas.

In their research^31^, the authors propose a Deep Neural Network (DNN) model along with image pre-processing techniques to automate the extraction of crucial features from lung images. This automated detection of COVID-19 from digital images holds the potential to expedite COVID-19 diagnoses and address the scarcity of clinicians in rural areas^32^. The DNN model they propose is based on a Convolutional Neural Network (CNN) architecture, which effectively extracts significant features from enhanced lung images, resulting in superior detection accuracy. Additionally, they introduce a framework called CovFrameNet, which encompasses a pipeline of image pre-processing methods, the DNN model, and a result verification technique. Initially, the researchers apply image pre-processing techniques to eliminate undesired distortions from CT and chest X-ray images, thereby enhancing the overall quality of the images. Considering the diversity and variations in image sizes within the database, they initially scale down the images to a standardized size of 220 × 220 pixels. To address sample distortions, they employ the Gaussian technique. Prior to segmenting the images, morphological operators such as opening and closing operations are applied to preprocess the samples. This step enables the extraction of image fragments that aid in representing and defining the shape of the region of interest.

To enhance the recognition of significant image features that facilitate feature extraction and yield meaningful outcomes, the study employs the thresholding segmentation technique. This technique ensures precise segmentation of the images, resulting in more specific regions of interest. The authors also utilize dilation to enlarge the foreground of the image and focus on a particular region within the background area. Moreover, they aim to identify the precise background area by employing a distance transform operation. This operation represents a binary image, where each pixel’s value is replaced with its distance from the closest background pixel. Consequently, the image is segmented into object and background regions. The thresholding segmentation process is accomplished using global thresholding, which employs a constant threshold value (T) for the entire image, generating the output image from the original one.

In their experiments, the authors employ two optimizers, namely Adam and SGD, independently, to examine the proposed approach^21^. One limitation observed during the study is the issue of memory constraint due to the considerable number of parameters in the model, which requires a significant amount of memory. However, the authors suggest that this challenge can be addressed by optimizing hyperparameters, thereby eliminating operations that do not contribute significantly to the algorithm's performance.

### Transfer learning

Transfer learning is a machine learning technique that involves utilizing a pre-trained model, trained on a large dataset, to initialize the weights of another model for solving either the same problem or a different problem domain^33^. As discussed in^21^, transfer learning offers significant advantages such as improved performance and reduced training time. Pre-trained models are typically trained on extensive datasets like ImageNet-21 k^16^. In the initial layers, convolutional neural networks (CNNs) learn basic features such as edge detection and gradually progress to learning more specific features closer to the classification layer. Since pre-trained models have already learned these basic features from massive datasets, there is no need for models using transfer learning to relearn them. This leads to a reduction in training time and an improvement in performance.

In the study presented in^34^, five pre-trained DNN models—ResNet50, ResNet101, ResNet152, InceptionV3, and Inception-ResNetV2—were proposed for detecting COVID-19-infected patients in the CXR dataset, which consists of four classes: COVID-19, Normal, viral Pneumonia, and bacterial Pneumonia. The results indicated that the pre-trained ResNet-50 model outperformed the other four models in terms of classification accuracy^34^. The reported performance results for ResNet-50 were 96.1% for Dataset-1, 99.5% for Dataset-2, and 99.7% for Dataset-3. As mentioned earlier, DNNs learn general features like edge detection in the upper convolutional layers and more dataset-specific features in the lower layers. Transfer learning allows other models to leverage this general feature knowledge, reducing the need to learn these features from scratch. Even with smaller datasets, transfer learning with optimal weight transfer leads to performance gains. The results presented in^34^ were further enhanced by transfer learning, compensating for the limited data available. Increasing the data and testing it across various centers could potentially enhance the performance of the five proposed models.

In another study described in^18^, the authors proposed a mechanism that combines three DNNs—VGG-16, ResNet-50, and Xception network—to create an effective COVID-19 classification model using CT images. The three models were initialized with optimal weights from pre-trained models. The hybrid model, based on transfer learning, achieved a classification accuracy of 98.79% and an F1-score of 0.99. The model developed in^18^ demonstrated promising results in accurate COVID-19 CT scan screening, suggesting its potential as a diagnostic tool for leading clinical specialists. The success of their results can be attributed to the ensemble learning strategy employed, which created a powerful COVID-19 prediction model. The authors used a stacking ensemble, which proved to be the most suitable approach for this task. By drawing inspiration from previous research studies^5,6^, they established their methodological approach.

The study introduced a modified version of ResNet^11^ called ResNet-v2^18^. Instead of using Batch normalization^4^, the authors employed Group normalization^35^ and applied weight standardization^36^ to all convolutional layers. Transfer learning was also incorporated, initializing the proposed model's weights using CIFAR-10^37^, ILSVRC-2012^15^, and ImageNet-21 k^16^ datasets for ResNet-v2. The proposed model in^18^ achieved a 99.2% accuracy rate in detecting COVID-19 cases. According to the authors, their model outperformed the neural architecture search model in all discussed measures. Despite limited data, the model performed satisfactorily, suggesting its applicability even in scenarios where large and diverse datasets are not readily available. Furthermore, the authors employed the Grad-CAM visualization technique to enhance the interpretability of the suggested deep learning model, enabling a better understanding of its functionality.

Hybrid models combine multiple models to perform a single task. In^38^, a study proposes a combination of Convolutional Neural Networks (CNN) for feature extraction and Long Short-Term Memory (LSTM) for classifying the extracted features from the CNN layers. The proposed model achieved accuracy, AUC, specificity, sensitivity, and F1-score of 99.4%, 99.9%, 99.2%, 99.3%, and 98.9%, respectively. The success of the proposed model lies in its hybrid architecture. However, the study^38^ identified several limitations. Firstly, the training data was relatively small, and increasing its size would help assess the generalizability of the established model. Secondly, the model needs to account for other perspectives of chest X-ray (CXR), such as the anterior–posterior (AP) view, as it currently focuses exclusively on the posterior-anterior (PA) image of CXR. Thirdly, accurate classification of COVID-19 images with multiple disease symptoms is required. Lastly, the performance of the suggested method needs to be compared to that of radiologists for more reliable results.

In^39^, the authors utilized a chest CT dataset as it is valuable for detecting lung disorders, including Pneumonia. They proposed a hybrid model that extracted features using models such as AlexNet, ResNet-18, ResNet-50, Inception-v3, Densenet-201, Inceptionresnet-v2, MobileNet-v2, and GoogleNet. These extracted features were then fed into the classification layer, and various machine learning (ML) algorithms, including Support Vector Machine, K-Nearest Neighbors, Naive Bayes, and Decision Tree, were employed for the classification task. The authors aimed to enhance the model's performance by utilizing Bayesian optimization to set optimal hyperparameters for the ML algorithms. Additionally, they incorporated an Artificial Neural Network (ANN) for image segmentation, allowing the CNN models to extract important features from the segmented area. Data augmentation was also performed on the dataset to improve the model's performance, as it has been widely reported to significantly enhance network performance^40^.

The study in^41^ highlighted the limited usage of optimization techniques to enhance the prediction performance of networks. Motivated by this observation, we dedicated time to hyperparameter optimization and conducted an experiment specifically focused on obtaining an optimal set of hyperparameters for both CNN and XGBoost models. The significance of optimized hyperparameters in improving the performance of machine learning (ML) models was emphasized in^39^, further motivating our pursuit of hyperparameter optimization. Additionally, the importance of hyperparameter optimization was mentioned in^31^ regarding memory issues caused by not optimizing hyperparameters.

The driving force behind this work stemmed from the realization of a lack of hybrid models specifically tailored for COVID-19, particularly those combining CNN and XGBoost models. XGBoost is recognized as one of the top-performing ML algorithms, as evidenced by its success in Kaggle image classification/regression competitions, where many past winners incorporated XGBoost either in their solutions or as a standalone algorithm, without forming a hybrid structure^42^. There is limited existing literature on the application of XGBoost in the context of COVID-19, and we aim to make a significant contribution to the fight against COVID-19 by integrating the XGBoost ML algorithm into our research. Furthermore, we acknowledge the effectiveness of CNNs, specifically VGG-16 and VGG-19, in developing classification models for COVID-19, as demonstrated in^24^. Therefore, our work aims to enhance the performance of our models through thorough hyperparameter optimization.

## Methodology

In this section the proposed methodology is detailed in the following sub-sections.

### The CNN architecture

**Figure 1:**
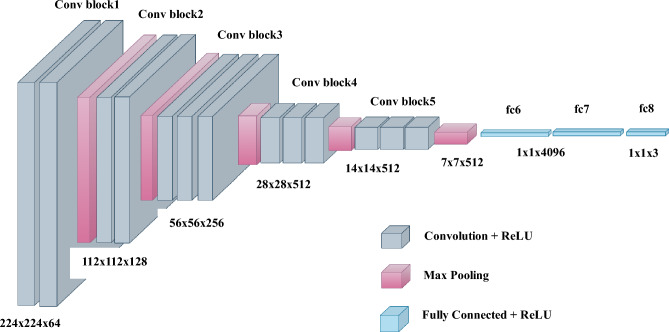
The CNN architecture.

Color images are represented as three-dimensional objects, with dimensions of height, width, and depth. Convolution operators, commonly used in image processing, expect input images to have these three dimensions. The filters used in convolution operations are typically square-faced cuboids, with a single parameter representing their size (height and width). During a convolution operation, the filter multiplies the pixels elementwise for each image patch, and the resulting output is the sum of these multiplications. However, since filter sizes may not always be factors of the input image's height or width, there can be regions of the image that are not covered by the filter, causing a border effect problem. Padding is a technique used to address this issue. Same padding is one type of padding where extra pixels are added along the image's borders to ensure that the dimensions of the output image match the dimensions of the input image. On the other hand, Valid padding is another type where the regions of the image not covered by the filter are ignored. The stride refers to the number of pixels by which the kernel (filter) moves horizontally during its slide along the image. A stride of 2, for example, reduces the size of the output image to half the size of the input image, while a stride of 1 results in overlapping windows. Pooling operations, such as maximum and average pooling, are used for downsampling the image using an untrainable kernel. A stride of 2 is often specified in pooling operations to achieve downsampling. Pooling also acts as a filter, introducing tolerance to convolutional neural networks (CNNs) by allowing neighboring pixel values to influence the values in the filter maps. While CNNs are generally robust to minor transformations, such as rotation or orientation differences, their tolerance has limitations. Major transformations may still affect their performance. Figure 1 illustrates the architecture of a CNN. The CNN's architecture is shown in Fig. 1.

### The hybrid CNN-XGBoost model and stand-alone CNN model

Hybrid CNN is an innovative approach that combines DNN models and/or ML models to divide the task responsibility into two parts. Typically, a DNN model like CNN is used to extract features from the dataset, while the ML model handles the classification aspect. This technique leverages the strengths of both DL and ML, overcoming their limitations and providing more accurate and computationally efficient solutions. In our work, we integrated the powerful CNN model, VGG-16, with the XGBoost ML model. In the following section, we provide justification for combining CNN with XGBoost.

XGBoost is a distributed gradient boosting library known for its effectiveness, adaptability, and portability. It utilizes the Gradient Boosting framework to develop machine learning algorithms, offering parallel tree boosting (GBDT/GBM) to solve various data science problems rapidly and accurately^43^. This robust algorithm, developed by Tianqi Chen^43^, gained significant attention in the ML community, particularly in competitions like Kaggle. Winning solutions in these competitions have either incorporated the XGBoost algorithm or relied solely on it^42^. XGBoost was designed for speed and performance by utilizing gradient-boosted decision trees^43^ and optimizing memory and hardware resources during training. It efficiently utilizes all available cores on high-processing machines, resulting in exceptionally fast execution. Additionally, it allows the incorporation and tuning of regularization parameters to mitigate overfitting. XGBoost also has the unique ability to boost data that has already been added to the trained model^44^.

Our research aimed to develop a model for automated COVID-19 detection in lung CT images following instances from existing results^41,45,46^. Both proposed models were trained on the CovidxCT-2A dataset, and their performance metrics were evaluated on the Covidx-2A dataset. To establish a baseline comparative analysis, we compared the performance metrics of our models with other state-of-the-art models in the literature^24^. Consequently, a demonstration was deemed appropriate to validate our approach. The study focused on automating the hyperparameter selection for the CNN and XGBoost models using Bayesian optimization. This approach aimed to achieve satisfactory results from the two proposed models: the CNN-XGBoost model and the stand-alone CNN. We named the hybrid model “CovNetBoost”.

XGBoost encompasses an extensive range of hyperparameters, and manually setting them up can prove to be futile. We explored different methods for tuning these parameters to fully leverage the XGBoost model. After reviewing several notebooks on Kaggle, we chose Bayesian Optimization due to its popularity and effectiveness. XGBoost's strength lies in its ability to automatically adjust thousands of learnable parameters to identify patterns and regularities in the data. We selected a few parameters for optimization, which we will briefly discuss individually. The chosen parameters are as follows: eta, colsample_bytree, gamma, max_depth, min_child_weight, n_estimators, reg_alpha, reg_lambda, and subsample. Let's discuss the important hyperparameters for XGBoost and their respective roles:

- Eta: This parameter helps prevent overfitting by introducing a step size shrinkage during updates. By reducing the feature weights at each boosting step, eta promotes a more cautious boosting process, ultimately strengthening the XGBoost model.
- Colsample_bytree: Belonging to the column subsampling family of parameters, colsample_bytree determines the ratio of columns to be subsampled when building each tree. In other words, subsampling occurs once for every tree constructed.
- Gamma: Denoting the minimum loss reduction required to split a node, gamma influences the algorithm’s conservativeness. Setting a higher value for gamma makes the algorithm more cautious in creating new splits.
- Max_depth: Used as a control measure to prevent overfitting, max_depth limits the depth of the XGBoost model. A higher depth allows the model to learn specific relations to individual samples, which can lead to overfitting.
- Min_child_weight: This parameter specifies the minimum total weight of observations required in a child. By using higher values, the model is restricted from learning relations that are extremely specific to particular samples, thus preventing overfitting.
- N_estimators: Analogous to the number of epochs in a CNN, n_estimators represents the number of iterations the XGBoost model will undergo to learn the features from the data.
- Reg_alpha and Reg_lambda: These parameters enable regularization on weights using L1 and L2 regularization strategies, respectively. They help prevent overfitting by introducing penalties on large weight values.
- Subsample: Subsample determines the number of observations to be randomly sampled for each tree. For example, if the value is set to 0.5, XGBoost would randomly select 50% of the training data before building trees. Subsample contributes to preventing overfitting and enhancing model generalization.

**Figure 2:**
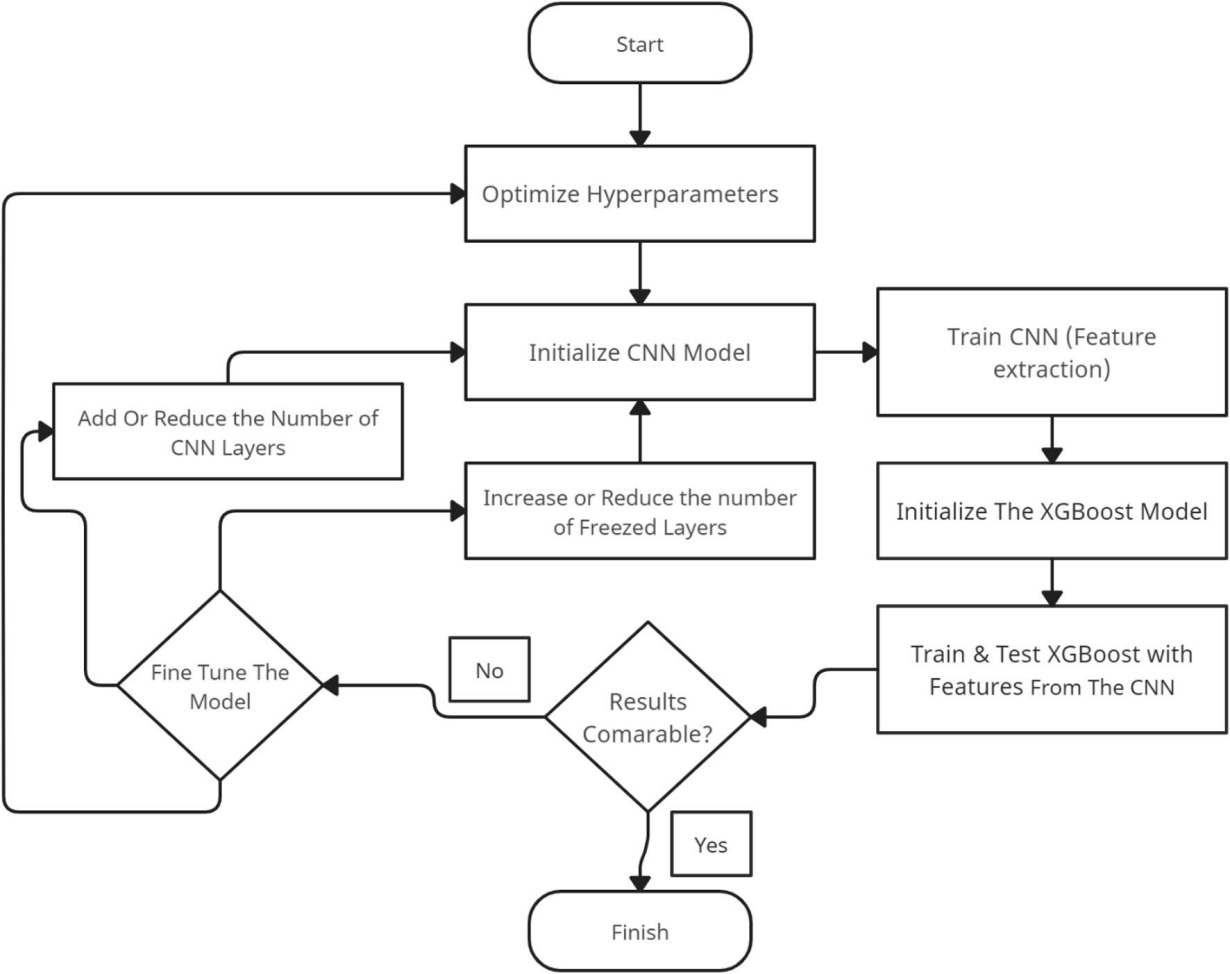
Flowchart showing the methodology steps we followed when experimenting with the hybrid CNN-XGBoost.

**Figure 3:**
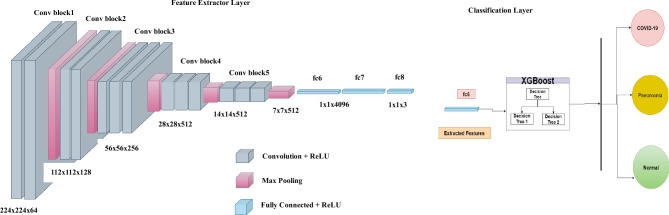
The illustration of the hybrid CNN-XGBoost model, also known as the CovNetBoost.

In Fig. 2, we illustrate the methodology steps followed during our experiments with the hybrid CNN-XGBoost approach. Additionally, Fig. 3 showcases the architecture of our proposed model, CovNetBoost.

**Figure 4:**
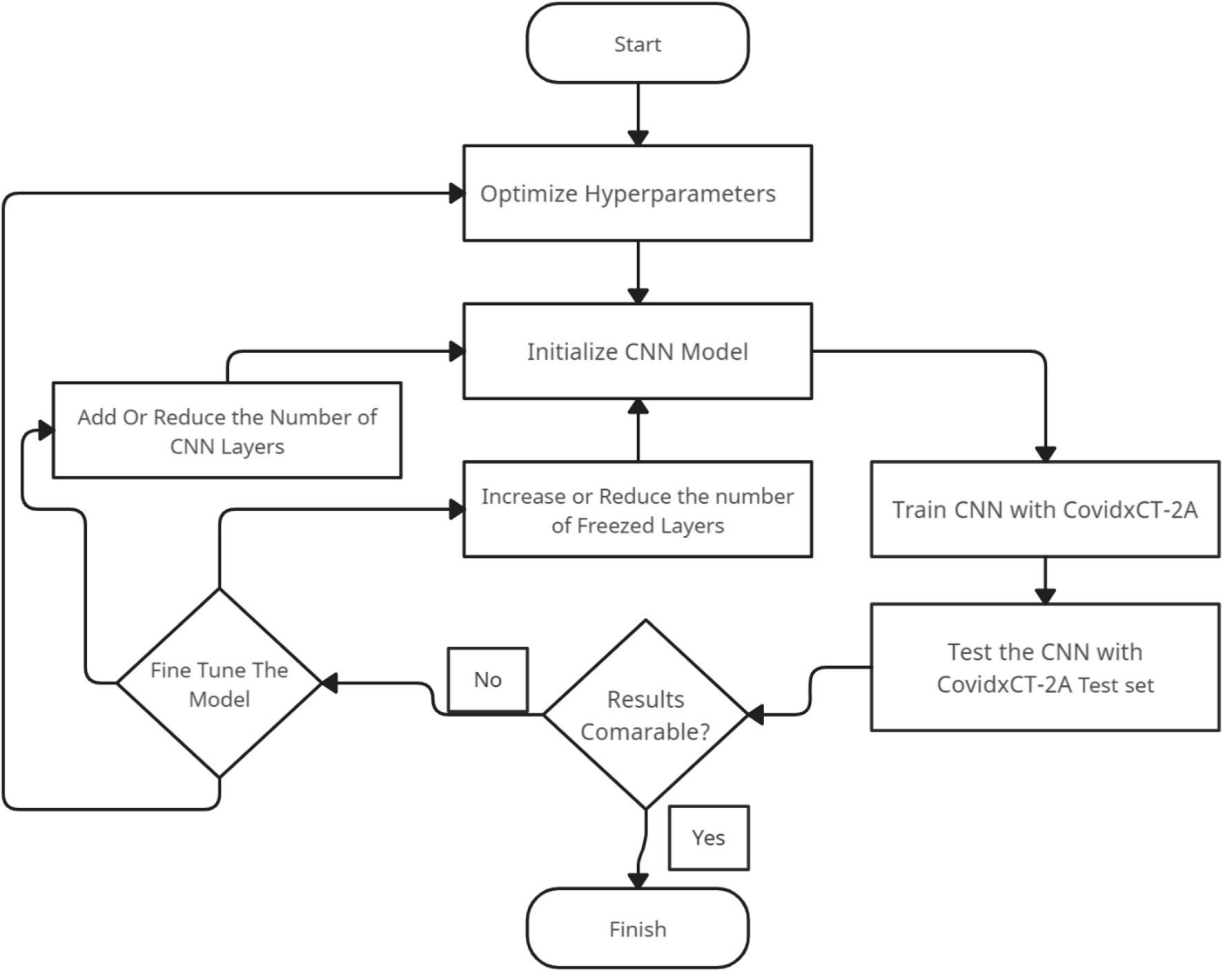
Flowchart showing the methodology we followed when experimenting with the stand-alone CNN.

**Figure 5:**
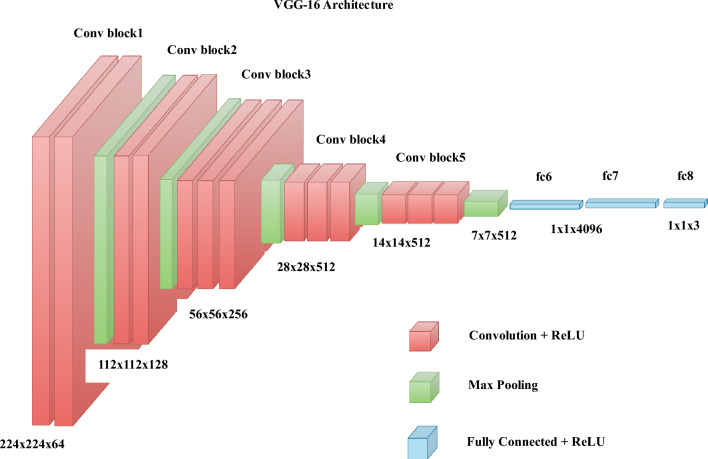
The stand-alone CNN architecture.

Figure 4 below illustrates the methodology steps we followed when experimenting with the stand-alone CNN model and Fig. 5 shows the stand-alone CNN architecture.

The design discussed in this section is implemented for experimental purposes fully described in the following section.

## Experimentation and evaluation metrics

The experimentation process described in the previous section revolves around utilizing a benchmark dataset and evaluating performance using widely recognized standard metrics. This section aims to provide a detailed elaboration on both aspects, thereby ensuring the reproducibility of the method proposed in this study.

To begin with, a benchmark dataset was employed as the foundation for conducting the experiments. The selection of this dataset was based on its widespread adoption and recognition within the research community. By employing a benchmark dataset, the study ensures that the results obtained can be compared and validated against other existing approaches, fostering a fair and objective evaluation of the proposed method. Furthermore, the performance of the method was rigorously evaluated using popular standard metrics. These metrics serve as quantitative measures to assess the effectiveness and efficiency of the proposed approach. By employing well-established metrics, the study ensures the credibility and reliability of the reported results. Moreover, the utilization of these metrics allows for straightforward comparisons with previous studies, enabling a comprehensive understanding of the method’s advancements and limitations. The explicit elaboration on the benchmark dataset and the standard metrics employed in the experimentation process contributes to the reproducibility of the proposed method. By providing clear details on these foundational elements, other researchers and practitioners can easily replicate and validate the study’s findings. This transparency not only facilitates the verification of the reported results but also encourages further advancements in the field by building upon the proposed methodology.

### Computational setup and dataset

The primary objective of the two proposed models is image classification. These models were developed using Python 3.7, incorporating popular machine learning libraries such as Keras and Tensorflow. All the experiments were conducted using Jupiter Notebook on Anaconda, utilizing an HP GeForce i7 12th Gen Intel(R) Core(TM) i7-12700H processor with 16.0 GB of installed RAM. The research focused on a specific problem domain, utilizing the CovidxCT-2A dataset for training and testing the models.

**Figure 6:**
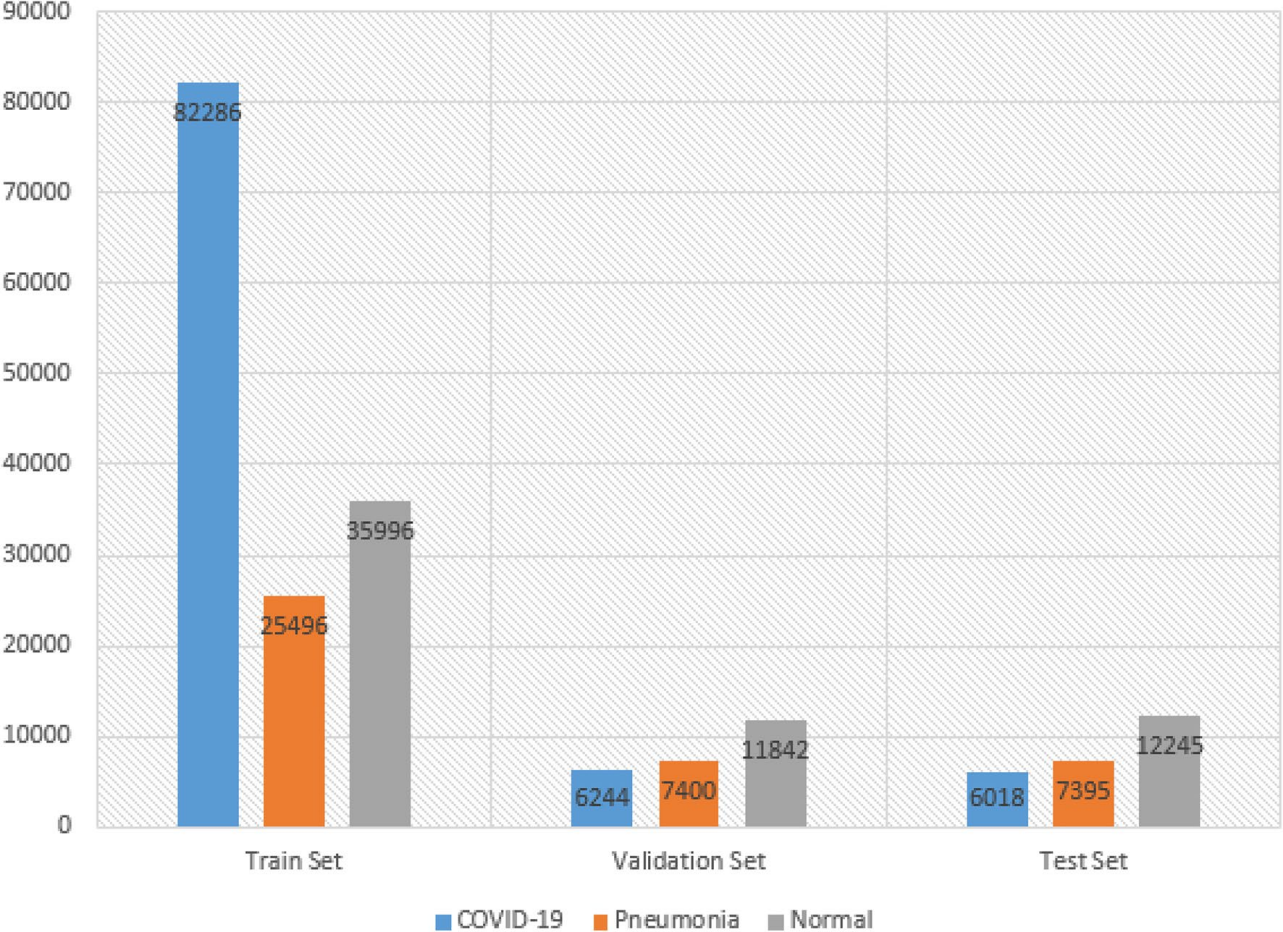
The distribution of the three classes on the training dataset.

**Figure 7:**
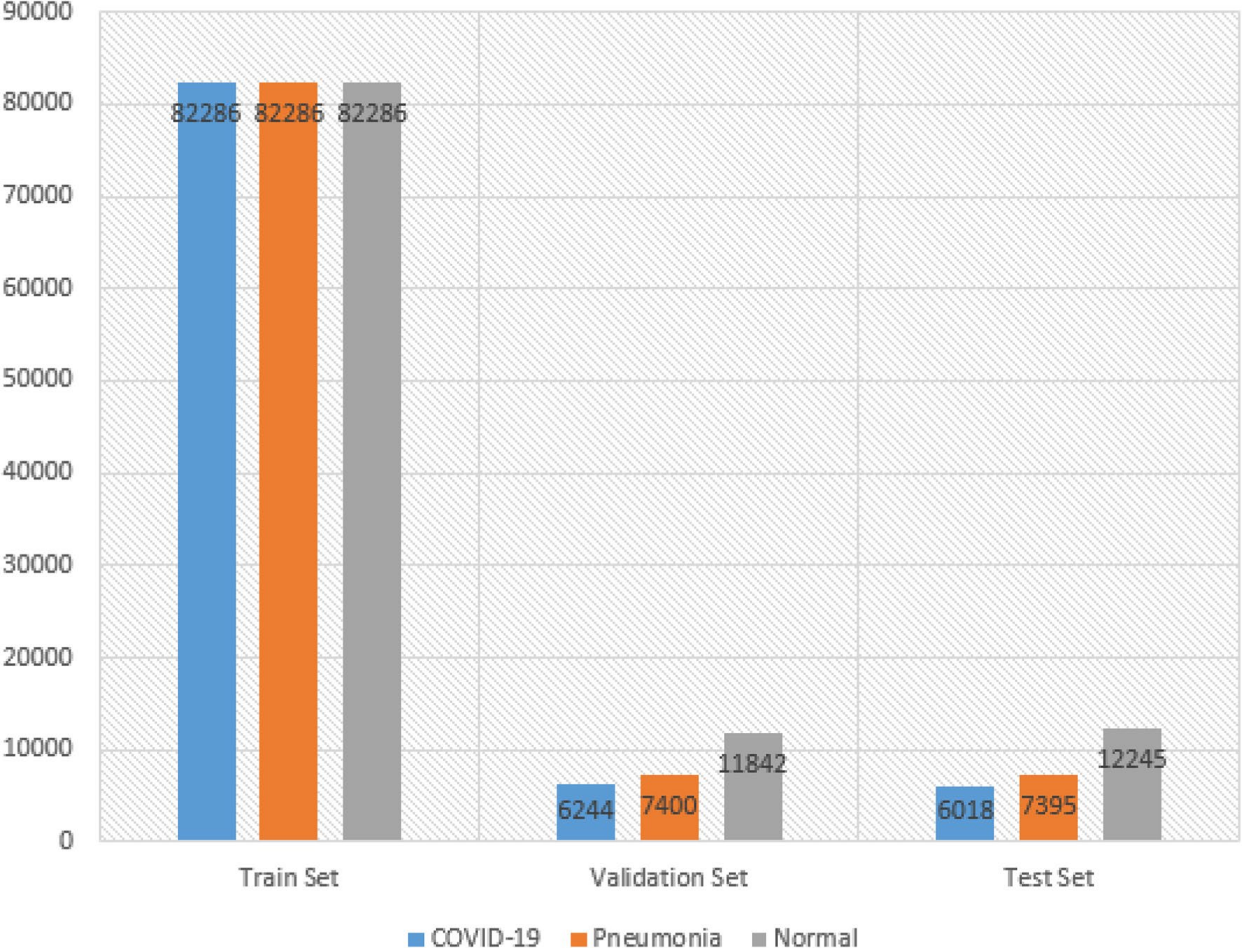
The distribution of the dataset after resampling has been applied to Pneumonia and Normal classes.

**Figure 8:**
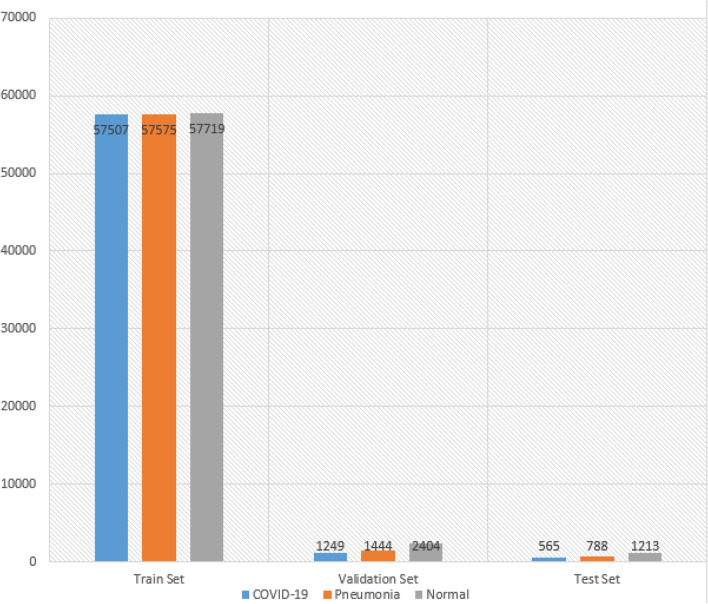
The distribution of data after sampling is 0.7 for the train set, 0.2 for the validation set and 0.1 for the test set*.*

The CovidxCT-2A dataset, referenced as^45^, consisted of 194,922 CT scans obtained from 3,745 patients across 15 different nations. The age of the patients ranged from 0 to 93, with a median age of 51. Each image in the dataset was associated with a specific class that had been verified by pathologists. The dataset comprised three classes: COVID-19, Pneumonia, and Normal. The COVID-19 class contained CT images of patients diagnosed with COVID-19, while the Pneumonia class included CT images of patients with Pneumonia unrelated to COVID-19. The Normal class encompassed CT images of patients without any lung disease. The inclusion of multiple nations in the dataset was part of a global cohort of patient cases collected by global organizations and initiatives, as outlined in^24^. Figures 6, 7 and 8 provide a visual representation of the distribution of our data across the three classes.

In Fig. 6, the distribution of data among the three classes is depicted, revealing a class imbalance issue. It is evident from Fig. 6 that the COVID-19 class has a higher number of instances compared to the Pneumonia and Normal classes. To address this problem, we aimed to balance the classes by augmenting the number of samples in the under-represented classes, namely Pneumonia and Normal, to match the number of instances in the COVID-19 class. The resampling method (upsampling technique) from the sklearn library was employed to achieve this. The code snippet 1 below shows how we up-sampled the two under-represented classes. The impact of the resampling method on the train set is illustrated in Fig. 7 below.
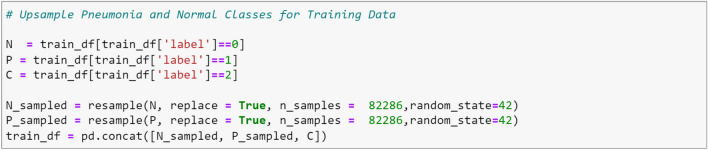


Code snippet 1: Resample method for adding more instances of Pneumonia and Normal classes.

In reference to^24^, the dataset was split into three subsets: 70% for the training set, 20% for the validation set, and 10% for the testing set. Figure 8 presents the outcome of applying the aforementioned sampling ratios to the data. The experiments were conducted utilizing specific hyperparameter configurations, as outlined in Tables 1 and 2. The results of each experiment were recorded at the conclusion of each run.

**Table 1:** Hyperparameters for the XGBoost model.

Params	Value
Eta	0.25
Colsample bytree	0.8598006602222718
Gamma	5.106825764829131
Max depth	11
Min child weight	3.0
N estimators	343
Reg alpha	66.0
Reg lambda	0.02657788124870091
Subsample	0.5

**Table 2:** Hyperparameters for the CNN model.

Params	Value
Solver	SGD
Max epochs	10
Batch size	16
Dense layer1	4096
Dense layer2	4096
Dropout1	0.1
Dropout2	0.9

In each of the five experiments conducted for the two proposed models, we will follow a specific procedure. Firstly, we will present the tables containing the performance metrics obtained from the experiments. These metrics will be used to evaluate the performance of the models. Afterward, we will plot a confusion matrix, which will visually display the True negatives (TN), True positives (TP), False positives (FP), and False negatives (FN) values obtained during each run. In order to interpret the results effectively, we will provide definitions for True negatives, True positives, False positives, and False negatives. These definitions will enable us to provide precise explanations about the performance measures utilized in this study. The performance measures we will be focusing on are Precision, Recall, F1-score, Specificity, and Accuracy. These metrics are calculated using the values from the confusion matrix mentioned above (TN, TP, FN, and FP). Let's briefly explain each of the confusion matrix values before delving into the experiments:

- True positive (TP): This indicates that the model has correctly classified an instance as belonging to a particular class. In other words, both the actual value and the predicted value align correctly.
- False Negative (FN): The model has made an incorrect classification, wherein it has predicted a different class instead of the actual class. For instance, if the model wrongly predicts Pneumonia or Normal class instead of COVID-19, it would be considered a false negative.
- False positive (FP): This refers to the instances where the model has incorrectly classified a sample as belonging to a specific class, while in reality, it belongs to another class. It quantifies the number of times the Positive class label has been misclassified.
- True negative (TN): This signifies that both the actual and predicted values align, correctly identifying instances that do not belong to the class in question.

Now, let's provide some information about the dataset used in this study. The dataset comprises 194,922 CT scans obtained from 3745 patients. This implies that there are multiple images belonging to the same patient within the dataset. To ensure accurate explanations, we will primarily focus on patient-based analysis. In other words, when discussing the results, we will often refer to misclassified images as misclassified patients. For example, if there are nine misclassified COVID-19 images, we will refer to them as nine misclassified COVID-19 patients.

### Performance measures

To evaluate the performance of the hybrid CNN-XGBoost and stand-alone CNN models, we conducted five simulations and averaged the results. The following performance metrics were utilized to measure the performance of each model: Accuracy (Acc), Precision (Pre), Specificity (Spec), Recall (Rec), and F1 score:

- Accuracy: The ratio of correctly classified instances over the entire number of instances. Equation (1) shows the formula for computing the accuracy metric:
  1 $$Accuracy= \frac{TP+TN}{TP+TN+FN+FP}.$$
- Precision: Measures the number of times the label of the Positive class has been incorrectly predicted as belonging to another class. Precision is calculated using Eq. (2) below:
  2 $$Precision= \frac{TP}{TP+FP}.$$
- Specificity: Measures the proportion of correctly labeled true negatives. Specificity is given by Eq. (3) below:
  3 $$Specificity= \frac{TN}{FP+TN}.$$
- Recall: measures how well our model predicts True Positives. Equation (4) below shows how the recall is calculated:
  4 $$Recall= \frac{TP}{TP+FN}.$$
- F1-score: Represents the balance between Recall and Precision. F1-score is calculated using the Eq. (5) below:
  5 $$F1= \frac{2*TP}{2*TP+FP+FN}.$$

The experimentation setup described in this section and the performance evaluation metrics is applied to analyze the hybrid model proposed in the study. More so, the above mentioned five performance metrics provide valuable insights into the capabilities and effectiveness of the hybrid CNN-XGBoost and stand-alone CNN models.

## Results and discussion

The optimal hyperparameter configurations for both the CNN model and XGBoost ML algorithm were determined through experimental exploration. We conducted various experiments to identify the set of hyperparameter values that yielded higher validation accuracy. These optimized hyperparameter values were then utilized in our experiments. The CovNetBoost, which is the hybrid model combining CNN and XGBoost, as well as the stand-alone CNN model, were trained on the CovidxCT-2A dataset. The dataset was split according to the instructions provided by the authors in^24^. Specifically, we allocated 70% of the data for training, 20% for validation, and 10% for testing.

The primary objective of these ten experiments was to achieve high classification accuracy for COVID-19, Pneumonia, and Normal cases. Additionally, we aimed to obtain elevated values for Precision, Recall, Specificity, and F1-score. These performance metrics are crucial in assessing the models’ effectiveness in correctly identifying and distinguishing between different classes. To ensure the reliability and consistency of the results, each proposed model was simulated in five separate runs. This approach allowed us to obtain correlated outputs and assess the stability of the models’ performance. After completing the simulations, the results were averaged across the five runs. We then compared these averaged results with other models presented in the existing literature, serving as a benchmark for evaluating the performance of our proposed models.

### Results of CovNetBoost and stand-alone CNN models

The results of the first experiment are summarized in Tables 3 and 4, which provide detailed information on precision, recall, F1-score, and specificity for each class. Let's focus on the Precision values outlined in the tables. Taking the Precision for the “Normal” class as an example, which is calculated to be 99.03%, it indicates that out of 1240 patients classified as Normal, the model accurately predicted 1225 of them. In other words, the model had 1225 true positive (TP) predictions for Normal patients. To calculate Precision, we divide TP by TP + FP. To determine the False Positive (FP) value, we need to refer to the corresponding column for the “Normal” class. In this case, we have two values: 3 and 9. These values represent instances where patients belonging to other classes (Pneumonia and COVID-19) were incorrectly classified as Normal. Therefore, we have FP = 3 (from Pneumonia) + 9 (from COVID-19). By understanding these values, we can provide a detailed and comprehensible explanation: In the case of the 'Normal' class, the Precision value of 99.03% signifies that out of 1240 patients classified as Normal, the model accurately predicted 1225 of them. This means that the model correctly identified 1225 Normal patients. To calculate Precision, we divide the number of true positive predictions (1225) by the sum of true positive predictions and false positive predictions. In this case, we observe the corresponding column for the 'Normal' class, where we find values of 3 and 9 for false positives, representing misclassifications from the Pneumonia and COVID-19 classes, respectively. By providing such a detailed explanation, we aim to make it easy for readers to understand and follow the evaluation process and the specific metrics used to assess the model's performance.

**Table 3:** Performance metrics are broken down for each class.

Class labels	Precision	Recall	F1-score	Specificity
Normal	0.9903	0.9879	0.9891	0.9910
Pneumonia	0.9862	0.9903	0.9882	0.9946
COVID-19	0.9737	0.9737	0.9737	0.9918

**Table 4:** Performance metrics for CovNetBoost.

Net	Pre	Rec	Spe	Acc	F1	MAvA	MAvG
CovNetBoost	98.34%	98.40%	99.25%	99.01%	98.37%	98.37%	98.52%

**Figure 9:**
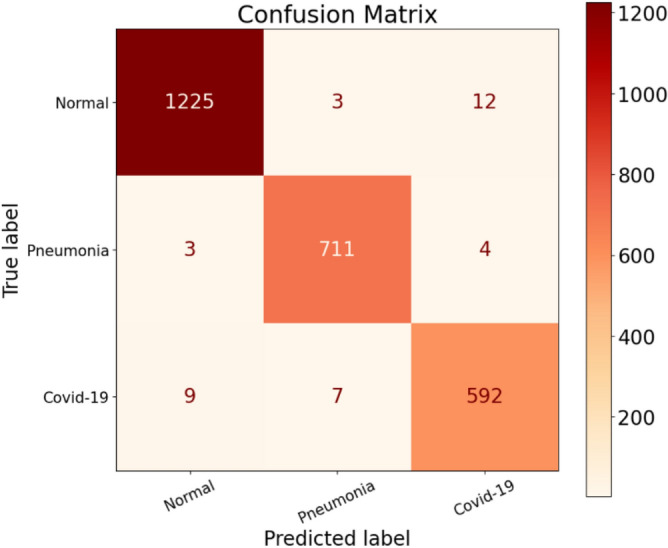
Confusion matrix for CovNetBoost.

During the testing phase, the model exhibited incorrect predictions for the Normal label a total of 12 times. Figure 9 illustrates these misclassifications, particularly in the Normal column (on the left). Upon closer examination, we discovered that out of the predicted Normal patients, 3 of them should have been classified as having Pneumonia, while 9 patients should have been identified as COVID-19 infected. Unfortunately, the model erroneously labeled these 9 patients as Normal individuals. These errors provide us with valuable insights. Firstly, it alerts us to the fact that 3 patients with Pneumonia were sent home under the impression that they belonged to the Normal class. Consequently, they may not seek appropriate treatment for their condition, unaware of their actual health status. Secondly, the misclassification of the 9 COVID-19 infected patients as Normal implies that they were not identified as carriers of the virus, despite actually being infected. This situation poses a significant risk as it could contribute to the further spread of the virus. Moreover, this places additional strain on healthcare facilities that are already overwhelmed. Therefore, these misclassifications emphasize the importance of refining the model’s accuracy to ensure appropriate categorization of patients and prevent potential repercussions such as increased virus transmission and added burden on healthcare systems.

During our analysis of the Pneumonia class, we discovered that the model made incorrect predictions for 10 patients, falsely identifying them as having Pneumonia when they were, in fact, not suffering from the condition. Out of these 10 misclassifications, three patients should have been labeled as Normal, while seven patients were incorrectly classified as having COVID-19 when they actually had Pneumonia. On a positive note, the model successfully detected 711 patients who were indeed suffering from Pneumonia. However, it did miss seven patients who had Pneumonia but were not detected during the process. These instances of misclassification provide us with valuable insights into the performance of our model. Turning our attention to the predictions related to the COVID-19 class, which is our primary focus, the misclassification of the seven patients as having Pneumonia when they were actually COVID-19 infected poses a significant danger to society. These individuals have the potential to exponentially increase the spread of the virus, as they may not receive the appropriate isolation and treatment necessary to contain the infection. To gain a comprehensive understanding of our model's performance, we delve deeper into the analysis of the results. Merely relying on accuracy as a performance metric can be misleading. Instead, we turn to the confusion matrix, which provides us with proper insight into the model's performance across different classes and metrics. By examining the performance of our model on a class-by-class basis, we gain an internal perspective on its efficacy. This approach allows us to obtain a more accurate evaluation compared to relying solely on overall accuracy. Hence, we divided our discussion to thoroughly assess the performance of our model, analyzing each class and metric separately.

Lastly, focusing on the COVID-19 class, we observed that out of the 592 COVID-19 patients, they were correctly identified as infected (true positives, TP). However, there were some errors made by the CovNetBoost model. It incorrectly predicted 12 patients as COVID-19 positive, when in fact they were labeled as normal (false positives, FP). Although this misclassification is undesirable, we believe it is better to classify patients as infected, even if they are not, rather than classifying them as normal and potentially missing actual COVID-19 cases.

On the other hand, there were nine patients who were labeled as normal but were actually infected with COVID-19 (false negatives, FN). These misclassified patients pose a risk as they could unknowingly spread the virus, contributing to the overall COVID-19 count. In this context, the 12 patients marked as COVID-19 cases but not infected can be seen as a precautionary measure since prevention is better than cure. Furthermore, the model also misclassified four patients as COVID-19 positive, whereas they had acquired pneumonia (FP for COVID-19 class, actual class Pneumonia). From our perspective, misclassifying a patient as COVID-19 positive is more acceptable than misclassifying them as normal or as having pneumonia. It is important to note that we are not implying the model's performance is satisfactory by making these misclassifications. However, compared to the potential consequences of missing COVID-19 cases, it is preferable to classify more patients as COVID-19 positive, even if they are not.

Moving forward, we will examine the Recall metric, which is crucial in our work to minimize false negatives (FN) as much as possible. Our primary objective is to avoid missing any COVID-19 patients since a higher number of undetected cases would contribute to a larger infected population. Upon evaluating the Recall values for the three classes, we found that the Pneumonia class exhibited the best Recall score, with 7 false negatives, compared to 15 for the Normal class and 16 for the COVID-19 class. Based on these results, we will prioritize the COVID-19 class in our classification efforts. In the COVID-19 row of our analysis, we identified nine patients who were classified as normal but were, in fact, infected by the virus. Additionally, in the Pneumonia column, seven patients were predicted to have pneumonia but were actually COVID-19 positive. The misclassification of these 16 patients poses a significant risk to the community, as the virus can spread rapidly. Ideally, our goal is to develop a model that minimizes these false negative values as much as possible. Specifically for COVID-19, we aim to achieve a false negative count of zero, indicating that no infected patients are missed in the classification process.

**Figure 10:**
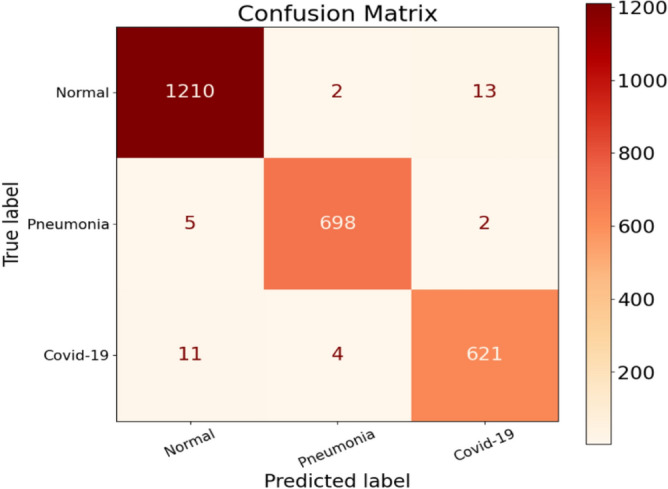
Confusion matrix for CovNetBoost.

The results of the second experiment are presented in Tables 5 and 6. Table 5 shows the Precision values for the Normal, Pneumonia, and COVID-19 classes, which are 98.69%, 99.15%, and 97.64%, respectively. Notably, the Precision value for COVID-19 is significantly lower, close to the experiment's overall Precision value of 1. This discrepancy prompts us to examine the misclassification errors related to false positives (FP) in more detail. Analyzing the confusion matrix for experiment 2 in Fig. 10, let’s begin with the Normal class. Out of 1225 patients labeled as Normal, only 1210 were correctly classified, resulting in 15 misclassification errors. Looking specifically at the false positives (FP) for the Normal class, there are 16 cases. Among these, five patients were classified as Normal but actually had Pneumonia, while 11 patients were classified as Normal but belonged to the COVID-19 class. This situation raises concerns, especially for rapidly spreading viruses like COVID-19. The misclassification of these 11 patients represents a significant number of missed COVID-19 infected cases.

**Table 5:** Performance metrics are broken down for each class.

Class labels	Precision	Recall	F1-score	Specificity
Normal	0.9869	0.9878	0.9874	0.9881
Pneumonia	0.9915	0.9901	0.9908	0.9968
COVID-19	0.9764	0.9764	0.9764	0.9922

**Table 6:** Performance metrics for CovNetBoost.

Net	Pre	Rec	Spe	Acc	F1	MAvA	MAvG
CovNetBoost	98.49%	98.48%	99.24%	99.04%	98.49%	98.48%	98.56%

On the other hand, the Precision for the Pneumonia class is the highest among the three classes, with only 6 false positive cases. In comparison, the Normal class had 16 false positives, and the COVID-19 class had 15. Among the misclassifications in the Pneumonia class, two patients were erroneously classified as having Pneumonia when they were actually normal, and four patients were misclassified as having Pneumonia when they were, in fact, infected with COVID-19. Lastly, focusing on the COVID-19 class, it demonstrates a low Precision score. There were two patients misclassified as COVID-19 infected when they had Pneumonia, and 13 patients misclassified as COVID-19 infected when they were actually normal. These findings highlight the misclassification errors in the experiment, particularly for the COVID-19 class. The low Precision in this class, coupled with the significant number of missed COVID-19 cases, is concerning, considering the rapid spread of the virus.

When examining the Recall values, it becomes evident that the Pneumonia class has a higher Recall compared to the COVID-19 and Normal classes. However, our primary focus lies on achieving high classification accuracy for the COVID-19 class. Taking a closer look at the COVID-19 class, we find that 621 patients were correctly classified as infected with COVID-19 (true positives, TP). The false negatives (FN) provide insight into how well our model performed in predicting this class. Figure 10 illustrates that four individuals were misclassified as belonging to the Pneumonia class, despite being infected with COVID-19. Additionally, we identified 11 patients who were misclassified as normal but were, in fact, infected by COVID-19. This misclassification is a matter of concern since a single individual can potentially spread the virus to an entire population. In the context of COVID-19, there is no room for misclassification. Furthermore, we discovered that 15 patients from the Pneumonia and Normal classes were incorrectly misclassified when they should have been classified as COVID-19 infected. This represents a significant number of missed COVID-19 cases by our model, highlighting its performance issues specifically regarding COVID-19 classification.

**Figure 11:**
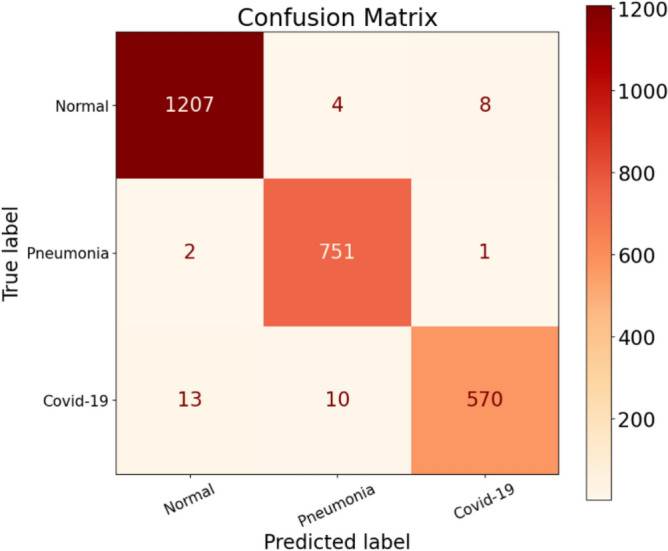
Confusion matrix for CovNetBoost.

Experiment 3 was carried out, and the results are presented in Tables 7 and 8. Examining Table 7, we observe that the Precision values for the Normal, Pneumonia, and COVID-19 classes are 98.77%, 98.17%, and 98.45%, respectively. This time, there is an improvement in the Precision rate for COVID-19 compared to the previous two experiments. The reduction in false positives (FP) for COVID-19 is a contributing factor, as it decreased significantly compared to 15 in experiment 2 and 16 in experiment 1. Analyzing the Normal class in Fig. 11, we find that the model incorrectly predicted two Pneumonia patients and 13 COVID-infected patients as normal cases. This misclassification is concerning, especially for COVID-19, as these 13 patients would leave the testing facility believing they are not infected when, in fact, they are. It is crucial to identify and isolate COVID-19 cases accurately to prevent further transmission.

**Table 7:** Performance metrics are broken down for each class.

Class labels	Precision	Recall	F1-score	Specificity
Normal	0.9877	0.9902	0.9889	0.9889
Pneumonia	0.9817	0.9960	0.9888	0.9923
COVID-19	0.9845	0.9612	0.9727	0.9954

**Table 8:** Performance metrics for CovNetBoost.

Net	Pre	Rec	Spe	Acc	F1	MAvA	MAvG
CovNetBoost	98.46%	98.24%	99.22%	99.01%	98.35%	98.35%	98.52%

Moving to the Pneumonia class, we observe that four patients were identified as having Pneumonia, but they were actually normal patients. Additionally, ten COVID-19 patients were misclassified as belonging to the Pneumonia class. Again, this is not an ideal prediction since these ten individuals could potentially contribute to an exponential increase in COVID-19 infection numbers. Lastly, when we consider the COVID-19 class, there is only one misclassification where a patient with pneumonia was identified as a COVID-19 case, and eight individuals identified as having COVID-19 were actually normal patients, as depicted in Fig. 11. From our perspective, these misclassification numbers are not too alarming. In this context, misclassifying a person as COVID-19 positive and isolating them is seen as a safety precaution. It is considered better to err on the side of caution by misclassifying a patient as COVID-19 positive rather than misclassifying them as normal or having pneumonia when they are actually infected with COVID-19. Overall, even a single misclassification, such as failing to identify a single infected patient, is deemed unacceptable in this case.

The Recall for the COVID-19 class remains relatively low compared to Pneumonia and Normal, mainly due to the significant number of false negatives (FNs), which amounts to 23. In contrast, Pneumonia has 3 FNs, and Normal has 12 FNs. This high number of FNs significantly affects the Recall score for COVID-19. Examining the Recall values, we find that the model predicted 10 COVID-19 patients as having acquired Pneumonia, incorrectly classifying their condition. Additionally, the model predicted the remaining 13 COVID-19 infected patients as individuals not suffering from any lung disease. This misclassification results in a total of 23 FNs, which is a relatively large number. Such misclassifications can lead to a considerable strain on healthcare resources due to the rise in COVID-19 infections. In summary, the low Recall for COVID-19, coupled with the significant number of FNs, indicates the model's difficulty in accurately identifying COVID-19 cases. These misclassifications have serious implications for healthcare systems, as they contribute to the spread of the virus and place additional strain on resources.

**Figure 12:**
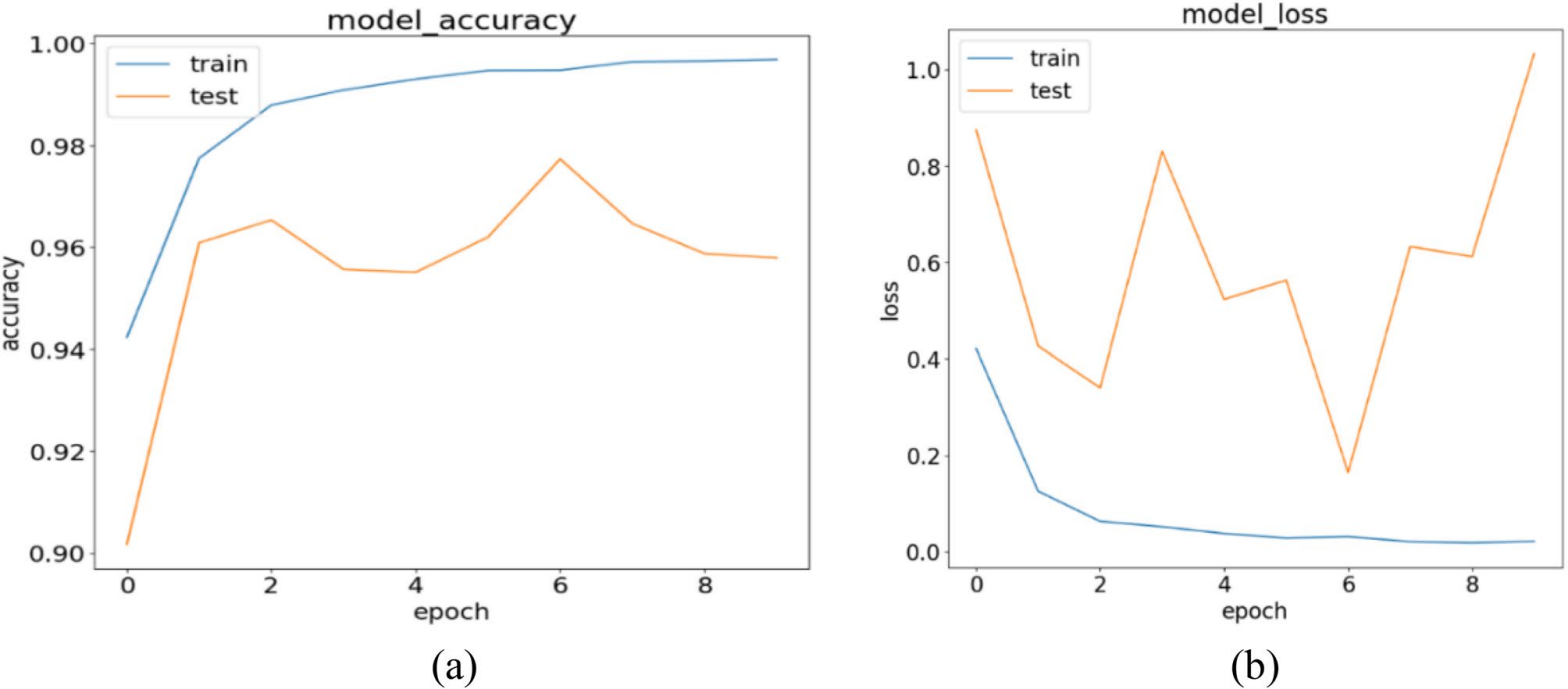
CNN’s training in terms of (**a**) validation accuracy and (**b**) validation loss.

The results obtained from the stand-alone CNN model for the three experiments are discussed in the following paragraphs. Examining Table 9, we focus on the Precision scores for the COVID-19 class, which is 96.25%. This indicates that out of all the tested COVID-19 infected patients, the model achieved a true positive (TP) count of 564. In other words, the model correctly classified 564 patients as belonging to the COVID-19 class but made incorrect predictions 22 times. Breaking down this number, the model identified 19 normal patients as COVID-19 infected and three pneumonia patients as having COVID-19. As a result, these 22 patients would be considered a risk to society, when in reality, they were not infected with COVID-19. Shifting focus to the Normal class, which has the highest number of patients among the three categories, it achieved a TP count of 1186. The model accurately classified 1186 out of 1226 normal patients, but it made four incorrect predictions by assigning the normal label to patients who were not normal. Table 9 highlights that the Normal class exhibits the highest Precision score, reaching 99.66%, compared to 96.23% for COVID-19 and 96.71% for Pneumonia. Table 10 provides a summary of the results obtained from the stand-alone CNN model. In Fig. 12a,b, we present the training accuracy and loss values of the trained CNN model.

**Table 9:** Performance metrics are broken down for each class.

Class labels	Precision	Recall	F1-score	Specificity
Normal	0.9966	0.9674	0.9818	0.9970
Pneumonia	0.9671	0.9961	0.9814	0.9855
COVID-19	0.9625	0.9843	0.9733	0.9890

**Table 10:** Performance metrics for the stand-alone CNN.

Net	Pre	Rec	Spe	Acc	F1	MAvA	MAvG
Stand-alone CNN	97.54%	98.26%	99.05%	98.65%	97.88%	97.89%	97.99%

This is supported by the low number of false positives (FPs) for the Normal class, which amounts to four, compared to 22 for COVID-19 and 26 for Pneumonia classes. Concerning the Normal class, the model made the incorrect prediction of four patients as normal, while they were actually infected with COVID-19. However, the model did not make any mistakes in misclassifying Pneumonia patients as patients without any disease. These positive results are notable, as in all five experiments conducted for the hybrid model, there was no instance of zero misclassification. Examining the Pneumonia class, which achieved a Precision rate of 96.71%, the model accurately predicted 764 out of 767 Pneumonia patients. However, it made 26 misclassifications by incorrectly assigning the Pneumonia label. Specifically, the model predicted 21 patients as having Pneumonia, whereas they were actually normal patients, and it classified five COVID-19 infected patients as having Pneumonia. Figure 12 illustrates the model's training progress based on (a) validation accuracy and (b) validation loss.

**Figure 13:**
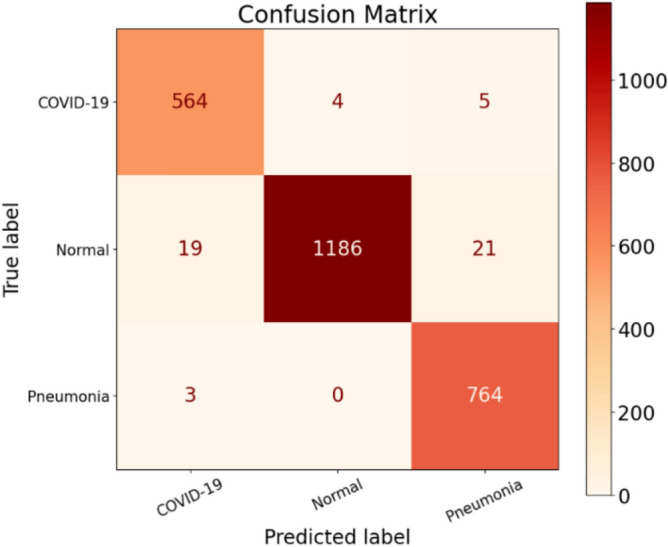
Confusion matrix for the stand-alone CNN model.

Analyzing the Recall scores for each of the three classes, we observe values of 96.74%, 99.61%, and 98.43% for Normal, Pneumonia, and COVID-19, respectively, as shown in Table 9. Notably, Pneumonia exhibits the highest Recall compared to Normal and COVID-19 classes. This is attributed to the Pneumonia class having the lowest number of false negatives (FNs), with a count of three, in contrast to nine FNs for COVID-19 and 40 FNs for the Normal class, as depicted in Fig. 13. When considering the Normal class, it achieved a high Precision score but yielded the lowest Recall score. The low Recall value is justified by the significant number of FNs, as the model misclassified 19 Normal patients as COVID-19 infected and 21 Normal patients as having Pneumonia. In regard to the COVID-19 class, the model generated nine FNs, which is the second lowest after Pneumonia. Out of a total of 573 patients, the model accurately predicted 564 COVID-19 cases but missed nine COVID-19 infected patients. Additionally, the model made the incorrect classification of four COVID-19 cases as Normal patients and five COVID-19 infected patients as having Pneumonia. These nine missed patients would go home believing they were not infected by COVID-19, posing a potential threat of spreading the virus to their close contacts. Lastly, Pneumonia achieved the highest Recall score of the three classes, with only three FNs, as shown in Fig. 13. The model performed well and did not make any prediction errors when classifying Pneumonia patients as Normal. The three FNs recorded for the Pneumonia class correspond to Pneumonia patients who were incorrectly predicted to be COVID-19 infected.

**Figure 14:**
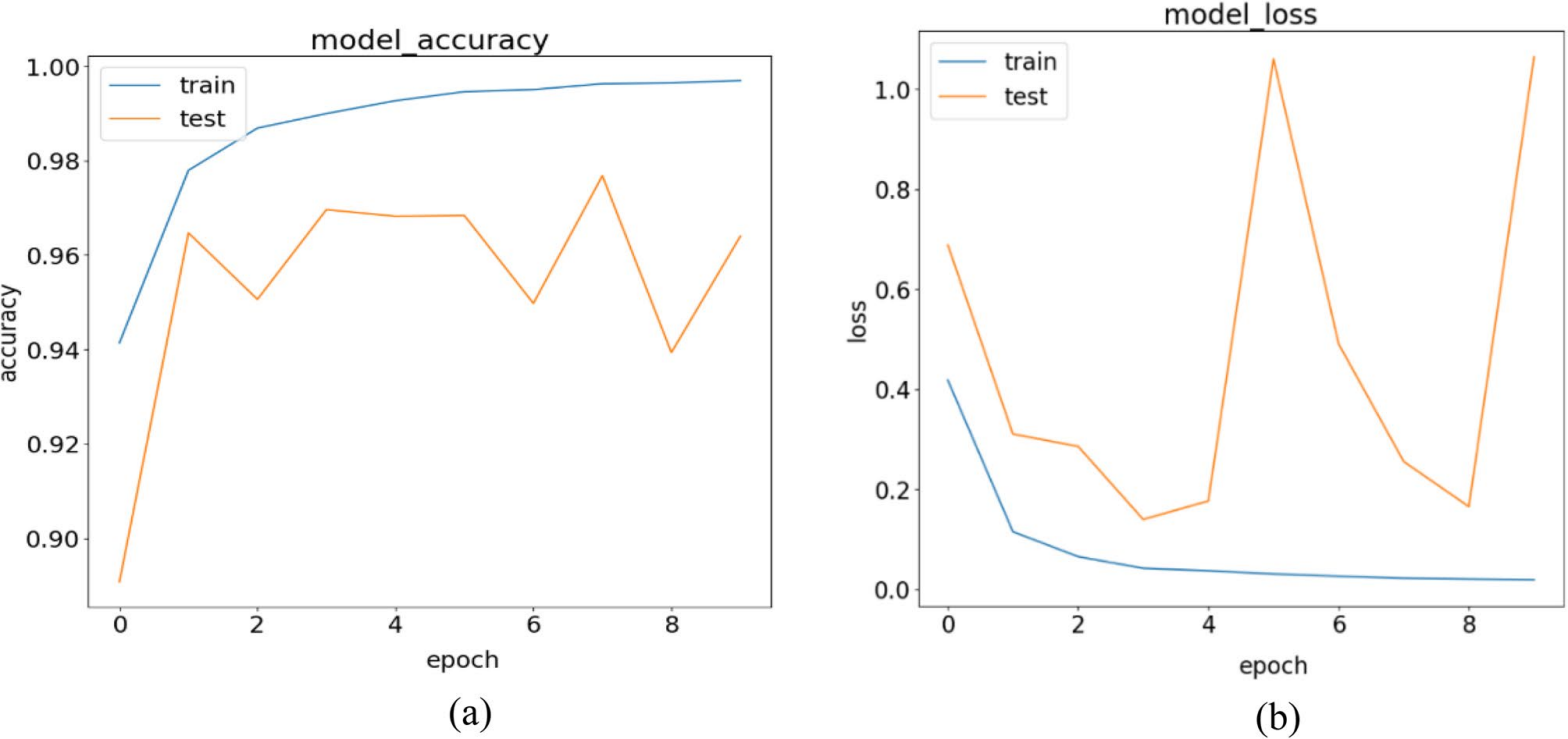
CNN training in terms of (**a**) validation accuracy and (**b**) validation loss.

The results of Experiment 2 for the stand-alone CNN model are presented in Tables 11 and 12, providing an overview of the class-based performances and performance metrics for the model. In Table 11, the Precision values for Normal, Pneumonia, and COVID-19 are 99.26%, 96.49%, and 98.68%, respectively. We observe an improvement in the Precision rate for COVID-19 compared to the previous experiment, which was 96.25%. Examining the Recall values for the three classes in Table 11, we find values of 98.13%, 100%, and 96.76% for Normal, Pneumonia, and COVID-19, respectively. Notably, the Pneumonia class achieves a perfect Recall score of 100%, with zero false negatives (FNs). The model successfully avoids any errors when classifying Pneumonia patients as either infected by COVID-19 or as Normal patients, which is the desired outcome for all three classes. The Normal class obtains the second-best Recall value, with the model accurately predicting (1210/1233) Normal patients. However, the Recall score for the Normal class is primarily affected by the number of FNs, which amounts to 23. Out of these 23 misclassifications that should have been predicted as Normal patients, the model incorrectly classified 8 patients as infected by COVID-19 and 15 patients as having Pneumonia. Lastly, focusing on the COVID-19 class, the model successfully predicted (598/618) COVID-19 patients but failed to detect 20 COVID-19-infected patients. It misclassified nine COVID-19 patients as Normal patients and incorrectly predicted 11 COVID-19 infected patients as having Pneumonia. Table 12 provides detailed performance metrics for the stand-alone CNN model, further evaluating its overall performance. In Fig. 14a,b, we show the trained CNN model's training accuracy and loss values.

**Table 11:** Performance metrics are broken down for each class.

Class labels	Precision	Recall	F1-score	Specificity
Normal	0.9926	0.9813	0.9869	0.9932
Pneumonia	0.9649	1.0000	0.9821	0.9917
COVID-19	0.9868	0.9676	0.9771	0.9959

**Table 12:** Performance metrics for the stand-alone CNN.

Net	Pre	Rec	Spe	Acc	F1	MAvA	MAvG
Stand-alone CNN	98.14%	98.30%	99.36%	98.88%	98.22%	98.21%	98.33%

**Figure 15:**
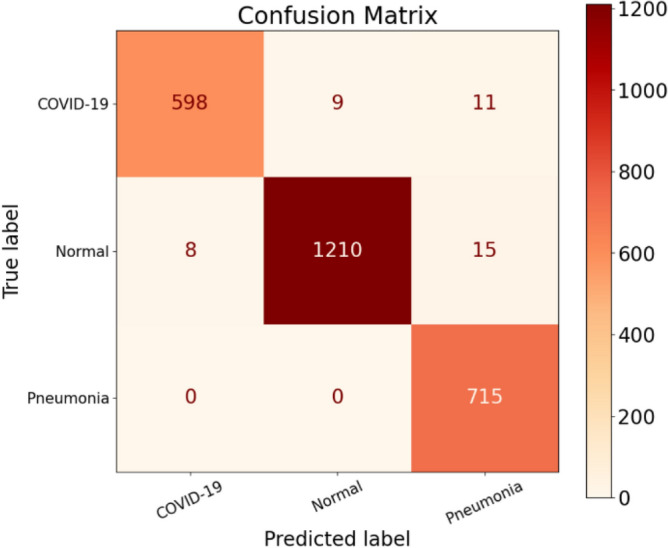
Confusion matrix for the stand-alone CNN model.

Upon analyzing the confusion matrix depicted in Fig. 15 for this experiment, we can observe a significant decrease in the number of false positives (FPs) compared to experiment 1. This reduction in FPs justifies the improved Precision score achieved in this experiment. In Table 11, we find that COVID-19 obtains the second-best Precision score among the three classes, with the Normal class achieving the highest Precision score of 99.26%. The COVID-19 class has 8 FPs, while the Normal class has 9 FPs. In contrast, the Pneumonia class records the highest number of FPs, which is 26. These misclassifications contribute to the lowest Precision score for the Pneumonia class. It's worth noting that a higher number of FPs for a class leads to a lower Precision rate. Further examining the FPs for the Pneumonia class, we discover that the model incorrectly classified 15 Normal patients and 11 COVID-19 infected patients as suffering from Pneumonia. These misclassifications contribute to the overall performance of the Pneumonia class and impact its Precision score negatively.

Analyzing the column corresponding to COVID-19 in Fig. 15, we can see that the model achieved a true positive (TP) value of 598. The TP represents the correctly classified COVID-19 patients. However, the model made eight false positive (FP) predictions, incorrectly labeling eight patients as COVID-19 positive. Out of these eight misclassifications, all were Normal patients, and the model did not make any errors in classifying Pneumonia patients as COVID-19 positive.

Examining the Normal class, the model accurately identified 1210 out of 1233 Normal patients, resulting in a high true positive rate. However, it incorrectly predicted the Normal label nine times, and in all cases, these misclassifications were COVID-19 infected patients that the model categorized as disease-free. This number of misclassifications is undesirable as it indicates a risk of missing infected COVID-19 patients and potentially contributing to the spread of the virus. Regarding the Precision discussion, looking at the Pneumonia column, we observe that all 715 Pneumonia patients were correctly classified by the model, resulting in a perfect true positive rate. However, this class has the highest number of false positives, with 26 instances. Out of these 26 misclassifications, 15 were Normal patients and 11 were COVID-19 infected patients, all predicted as having Pneumonia. Moving on to Experiment 3 conducted for the stand-alone CNN model, the results of the experiment are summarized in Tables 13 and 14, which provide detailed information on precision, recall, F1-score, and specificity for each class. The Precision values for the three classes are 99.28% for Normal, 97.46% for Pneumonia, and 98.17% for COVID-19, as shown in Table 13. In Fig. 12a,b, we show the training accuracy and loss values for the trained CNN model.

**Table 13:** Performance metrics are broken down for each class.

Class Labels	Precision	Recall	F1-score	Specificity
Normal	0.9928	0.9866	0.9897	0.9931
Pneumonia	0.9746	0.9900	0.9822	0.9904
COVID-19	0.9817	0.9768	0.9793	0.9944

**Table 14:** Performance metrics for the stand-alone CNN.

Net	Pre	Rec	Spe	Acc	F1	MAvA	MAvG
Stand-alone CNN	98.30%	98.45%	99.26%	99.01%	98.37%	98.37%	98.52%

**Figure 16:**
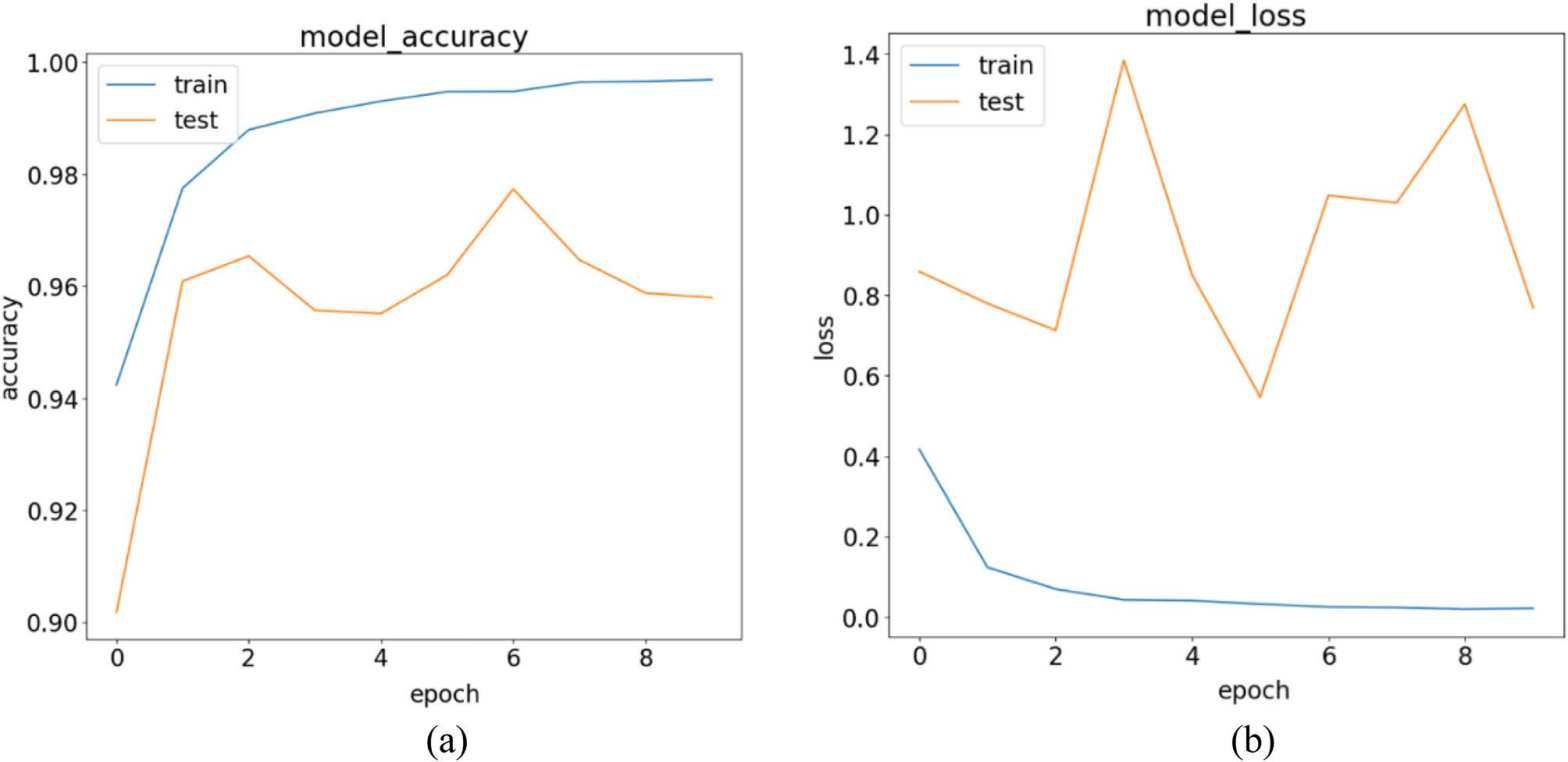
CNN training in terms of (**a**) validation accuracy and (**b**) validation loss.

**Figure 17:**
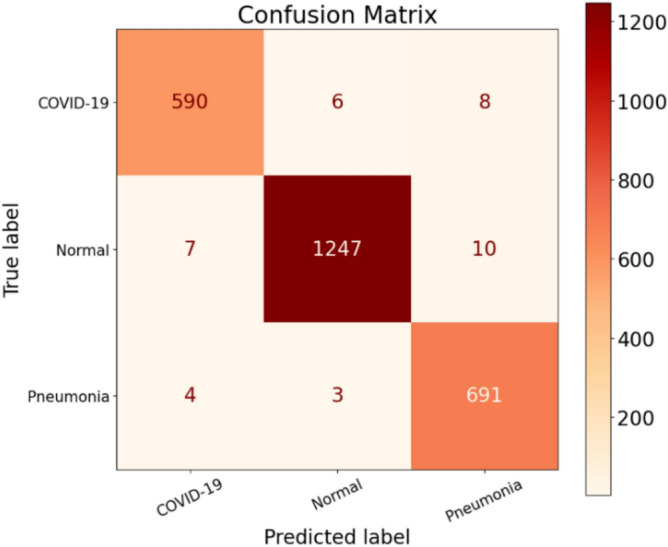
Confusion matrix for the stand-alone CNN model.

Figure 16 displays the training of the model, showcasing (a) validation accuracy and (b) validation loss. Examining the confusion matrix in Fig. 17 to analyze the Precision scores obtained, we observe that the COVID-19 class achieved a true positive (TP) value of 590. Out of the 604 COVID-19 patients, the model correctly classified 590, but made 11 false positive (FP) predictions. These misclassifications include seven Normal patients and four Pneumonia patients predicted as COVID-19 positive. Analyzing the Normal column in the confusion matrix, the model correctly predicted 1247 out of 1264 Normal patients, resulting in a high true positive rate. However, it made three false positive predictions for Pneumonia patients and six false positive predictions for COVID-19 patients, incorrectly labeling them as Normal. In the case of the Pneumonia class, out of 698 tested patients, the model accurately classified 691 as Pneumonia patients, resulting in a high true positive rate. However, it failed to detect seven Pneumonia patients (false negatives). The Pneumonia class also had 18 false positive predictions, including ten Normal patients and eight COVID-19 patients misclassified as having Pneumonia. Regarding Recall values, this experiment achieved 98.66% for the Normal class, 99.00% for the Pneumonia class, and 97.68% for the COVID-19 class, as shown in Table 13. These values indicate that the model performed well in correctly classifying Pneumonia patients compared to the COVID-19 and Normal classes.

The hybrid CNN model was further evaluated through two additional experiments, resulting in a total of five experiments for both the hybrid CNN and stand-alone models. In the subsequent sub-section, we will provide a comparative analysis of the performance metrics obtained for the hybrid CNN model. For the COVID-19 class, the hybrid CNN model had 14 false negatives (FNs), indicating that it failed to identify 14 COVID-19-infected patients. This is a significant number considering the potential impact on the rapid spread of infections. Among these missed patients, six were incorrectly classified as Normal cases, and eight were classified as Pneumonia cases. The Normal class exhibited 17 FNs, with the model correctly identifying 1247 out of 1264 Normal patients. Upon analyzing the 17 missed Normal patients, it was observed that ten were wrongly classified as Pneumonia cases, and seven were classified as COVID-19 cases. Regarding the Pneumonia class, there were seven FNs, and the model correctly predicted 691 out of 698 Pneumonia patients. Within the seven missed patients, three were incorrectly classified as Normal, and the remaining four were predicted as COVID-19 cases. This comprehensive analysis of the hybrid CNN model's performance highlights the strengths and weaknesses in its ability to accurately classify patients across the three classes.

### Comparing performance metrics of CovNetBoost

**Figure 18:**
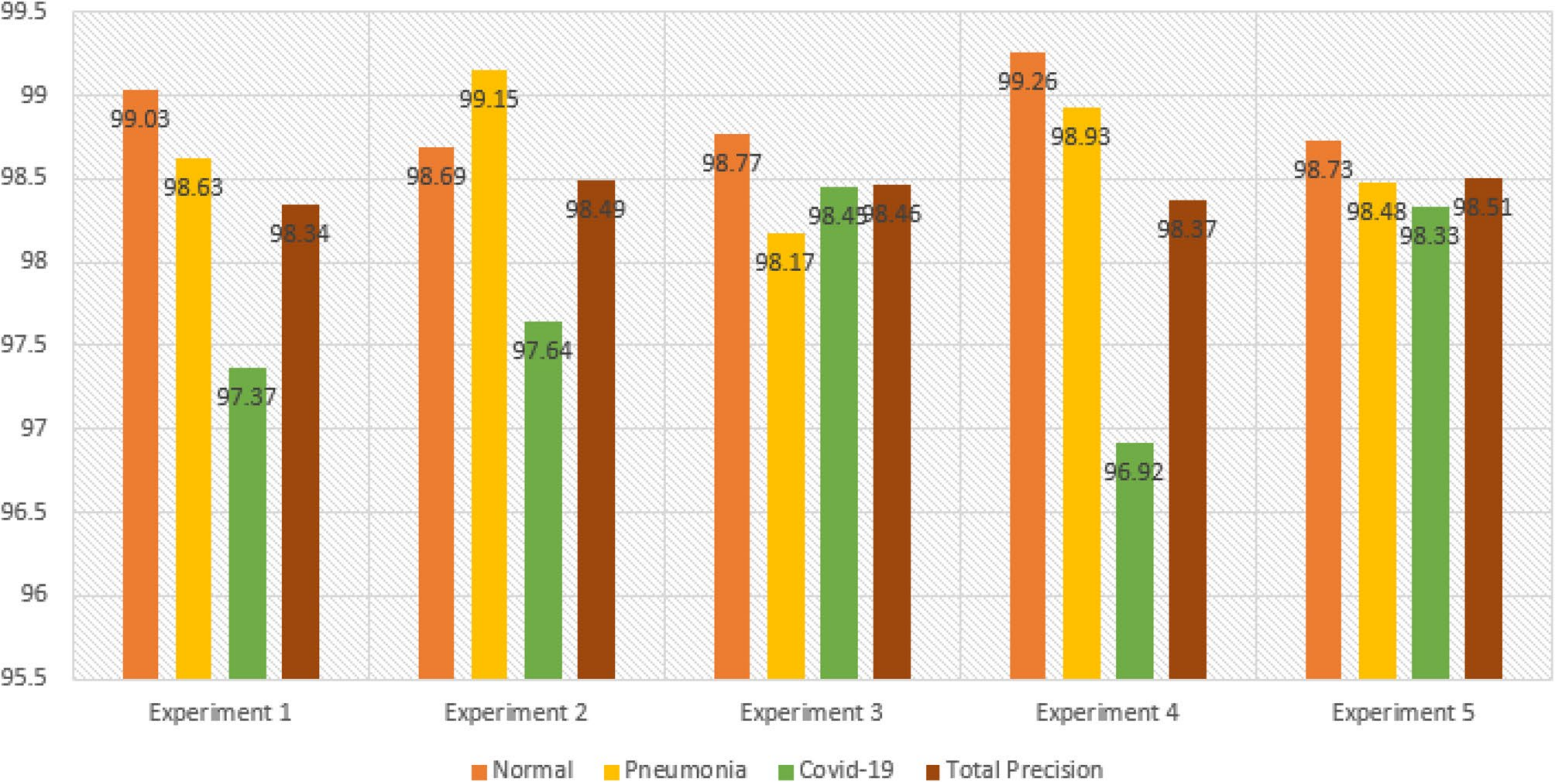
Comparing precision for the experiments conducted.

In this section, our goal is to present visualizations of the performance metrics obtained from the five experiments conducted on the hybrid model. We aim to provide a clearer interpretation of the discussions we presented for each experiment. In Fig. 18, Experiment 5 stands out with the highest overall Precision among the five experiments, achieving a value of 98.51%. This high Precision in Experiment 5 can be attributed to the closely correlated Precision values for all three classes, which range between 0.98 and 0.99. Following closely behind, Experiment 2 demonstrates the second-best overall Precision, reaching a value of 98.50%. Experiment 2 closely resembles Experiment 5, although it is affected by lower Precision in the COVID-19 class, which is 97.54%, compared to Experiment 5's 98.33%.

To provide an overview of all five conducted experiments, we measure Precision by considering the number of false positives (FPs) for each class. This involves tracking the instances where the model incorrectly predicted the label Pneumonia as belonging to either the COVID-19 or Normal class. A higher number of FPs results in a smaller Precision value. Let's now examine the FPs for each experiment. Both Experiment 5 and Experiment 2 have the same number of FPs, which is 37. Similarly, Experiment 1 and Experiment 3 share the same number of FPs, amounting to 38. In Fig. 20, representing Experiment 4, we observe a relatively low number of FPs, specifically 36. However, Experiment 4 is predominantly affected by the number of FPs in the COVID-19 class, which is 19. As a consequence, Experiment 4 exhibits the lowest Precision score for COVID-19, at 96.92%. Experiment 5 displays the best correlation among Precision values for the three classes, with the COVID-19 class achieving the lowest value of 98.33%. On the other hand, when examining the correlation among the values in the other experiments, they do not closely match. Experiment 1 records the lowest Precision from the COVID-19 class at 97.37%, while Experiment 2 reports the lowest value of 97.64%, also originating from the COVID-19 class. In Experiment 3, the lowest Precision value arises from the Pneumonia class, amounting to 98.45%. As mentioned earlier, Experiments 4 and 5 exhibit low FP values, which justifies why Experiment 5 achieves the highest Precision value. In Experiment 5, the model demonstrates almost equal Precision for all three classes, indicating a significant step towards the development of a high-performing model.

A model’s performance should be relatively consistent across all three classes and avoid excelling in one class while providing poor Precision in the others. Let's examine the best Precision obtained for each class across the experiments.

For the class Normal, Experiment 4 achieves the highest Precision with a value of 99.26%. This experiment also has the lowest number of false positives (FPs) for the Normal class among all five experiments, totaling 9. In comparison, Experiment 1, Experiment 2, Experiment 3, and Experiment 5 have 12, 16, 15, and 15 FPs respectively. Moving on to the class Pneumonia, Experiment 2 exhibits the best Precision with a value of 99.15%. Figure 18 shows that this experiment has 6 FPs. Experiment 1 has 10 FPs, Experiment 3 has 14 FPs, Experiment 4 has 8 FPs, and Experiment 5 has 12 FPs. Lastly, for the class COVID-19, Experiment 3 provides the highest Precision with a value of 98.45%.

**Figure 19:**
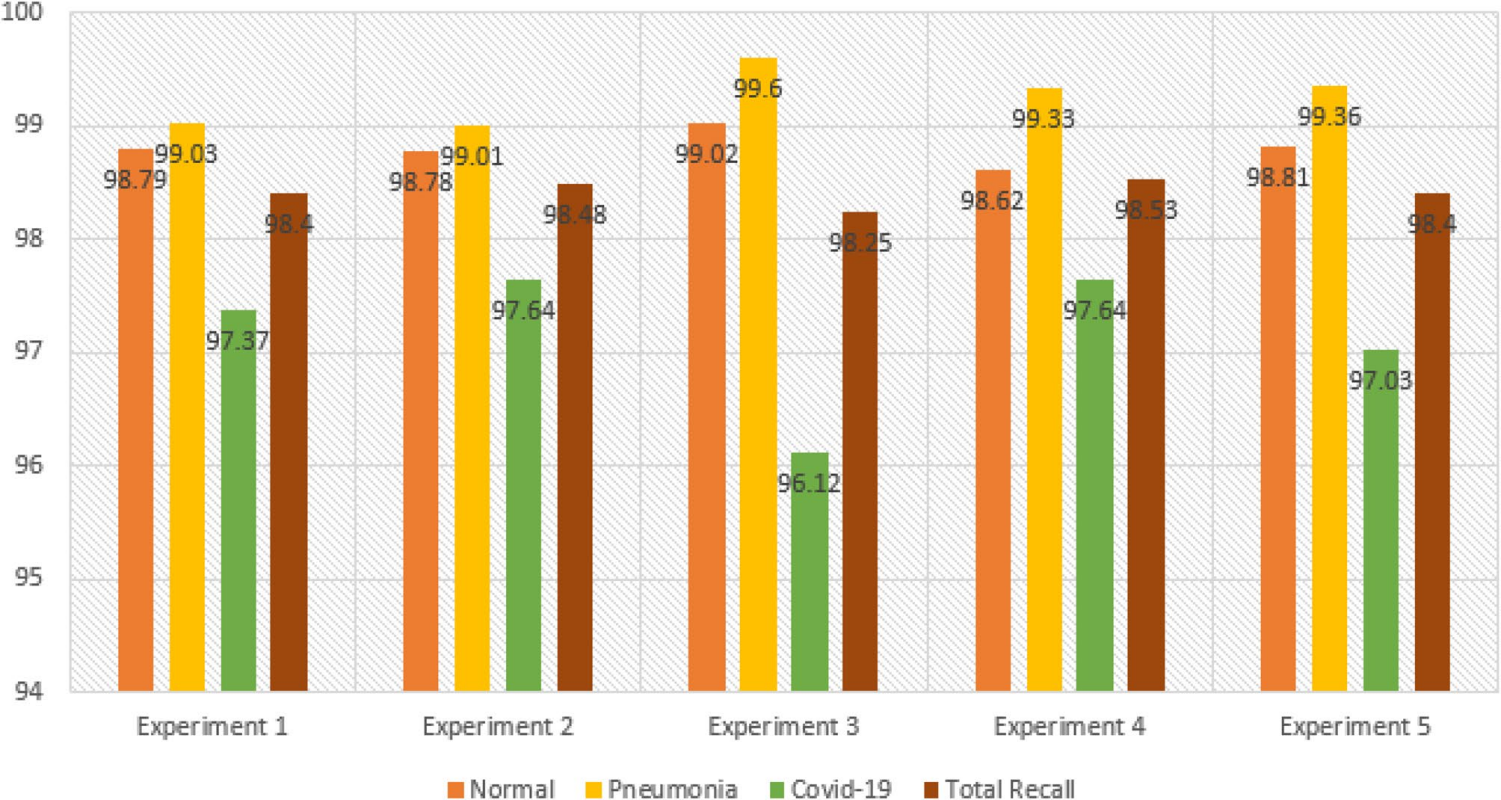
Comparing recall for the experiments conducted.

Across all five experiments, the number of FPs is as follows: Experiment 1 has 16 FPs, Experiment 2 has 15 FPs, Experiment 3 has 9 FPs, Experiment 4 has 19 FPs, and Experiment 5 has 10 FPs. Moving on to Fig. 19, it presents the comparative performances of the recall metric for the five conducted experiments. As mentioned earlier, achieving a high Recall value is crucial as it measures the number of false negatives (FNs) obtained from the model's predictions. It indicates how well our hybrid model performed in classifying patients into their correct categories. Experiment 4 demonstrates the best overall Recall value of 98.53%, while the lowest Recall value comes from Experiment 3, with a value of 98.25%. Experiment 5 achieves a high Recall score due to the best classification accuracy for COVID-19, reaching 97.64%. The high Recall in Experiment 5 is influenced by the close correlation of Recall values for all three classes. Similarly, Experiment 2 achieves an overall Recall value of 98.48%, which is the second-best across the five experiments. In Experiment 2, there are 14 FNs, meaning the model correctly identified 579 out of 593 patients with COVID-19 but missed 14 infected individuals. The Recall values for COVID-19 in both experiments are the same, despite having different samples. The number of samples is not crucial; what matters is accurately classifying the samples. This reflects a real-world scenario where the interest lies in correctly classifying patients tested on different days, regardless of the varying sample sizes. Regarding the number of missed COVID-19 patients in the remaining experiments, Experiment 1 has 16 misses, Experiment 3 has 23 misses, and Experiment 5 has 18 misses.

Experiment 3 yielded the highest Recall for Pneumonia, achieving an impressive value of 99.60%, while experiment 2 had the lowest Recall at 99.01%. Notably, the CovNetBoost model demonstrated exceptional performance in Pneumonia classification, as evidenced by its lowest value. Examining the number of missed Pneumonia patients in experiments with high and low Recall values, Fig. 19 reveals that experiment 3 only missed three Pneumonia patients, with the CovNetBoost model accurately identifying 751 out of 754 Pneumonia affected patients. On the other hand, experiment 2 missed seven Pneumonia patients, with the model correctly identifying 698 out of 705. In experiment 1, the Pneumonia classification accuracy stood at 99.03%, with the model failing to identify seven Pneumonia infected patients. Experiment 4 achieved a Pneumonia classification accuracy of 99.33%, correctly predicting 740 out of 745 patients but failing to identify five patients. Lastly, experiment 5 obtained a classification accuracy of 99.36% for Pneumonia, with the model achieving 777 out of 782 correct classifications and only missing five patients. Regarding the Recall for the Normal class, experiment 3 demonstrated the highest value at 99.02%, while experiment 4 had the lowest at 98.62%. In experiment 3, the model identified 1207 out of 1219 Normal patients but missed 12. In contrast, experiment 4 correctly predicted 1211 out of 1228 patients but failed to identify 17 Normal patients.

**Figure 20:**
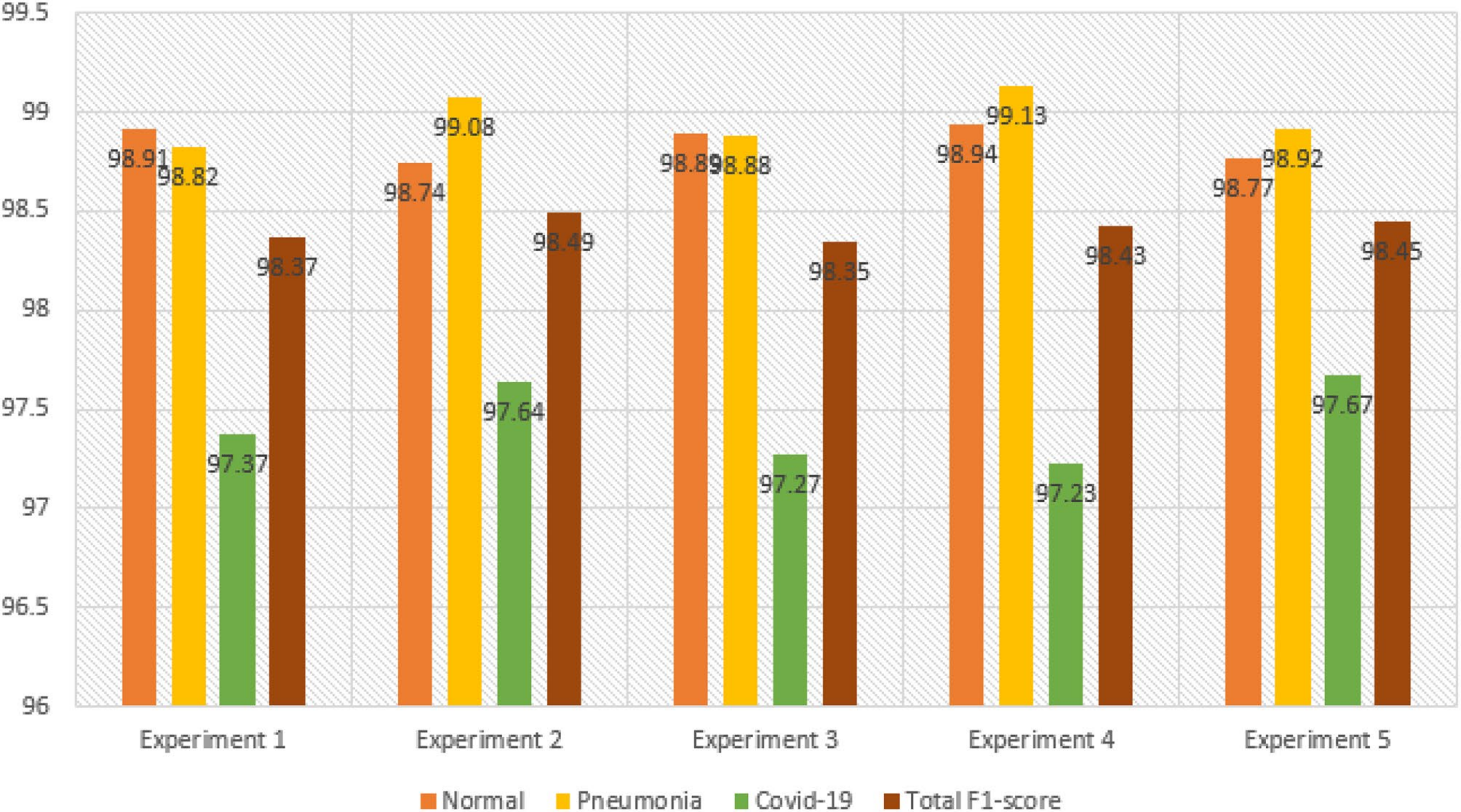
Comparing F1-score for the experiments conducted.

Comparing the F1-score performances across the five experiments as depicted in Fig. 20, experiment 2 yielded the best overall F1-score with a value of 98.49%, whereas experiment 3 had the lowest score at 98.35%. The F1-scores for the Pneumonia and Normal classes closely aligned across the five experiments, indicating that the model performed well in correctly identifying these two classes. However, the COVID-19 F1-score exhibited the most significant disparity, with experiment 5 achieving the highest score, closely followed by experiment 2 with a value of 97.64%. Experiment 4 boasted the highest Recall for the Pneumonia class at 99.13%, while experiment 1 had the lowest Recall at 98.82%. Finally, when considering the Normal class, experiment 4 exhibited the highest Recall at 98.94%, while experiment 2 had the lowest with a value of 98.74%.

**Figure 21:**
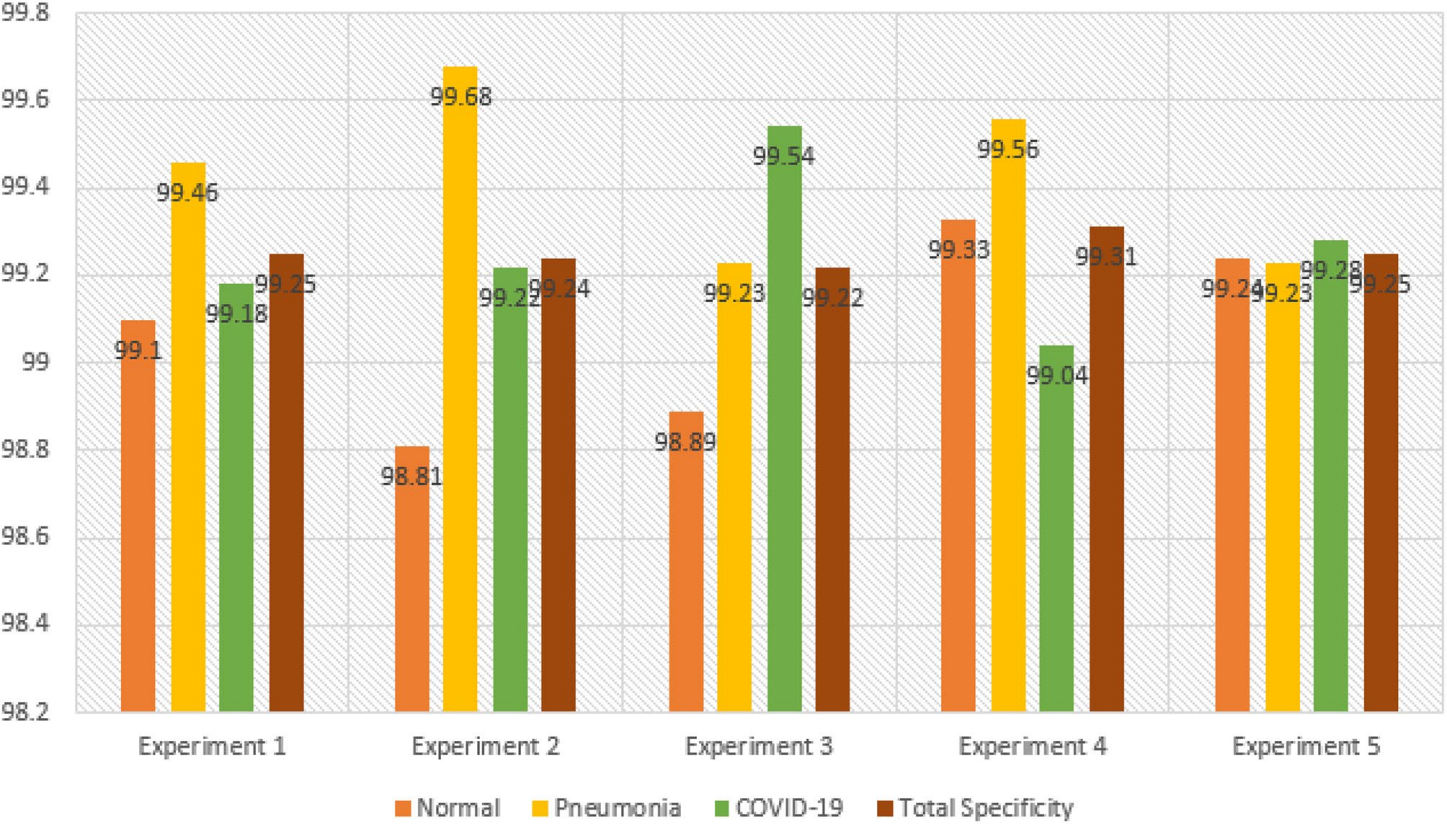
Comparing specificity for the experiments conducted.

Let’s now examine the Specificity metric across the five experiments, as depicted in Fig. 21. Specificity can be viewed as the complement of Recall. While Recall measures the classification rate of a specific class, Specificity evaluates how well a model classifies other classes. For example, when evaluating the Specificity of the Pneumonia class, we assess how accurately the model identifies classes other than Pneumonia. Specificity is influenced by the number of False Positives (FPs); the lower the FPs, the higher the Specificity value. In the case of the Pneumonia class, if there are zero FPs, we would achieve 100% Specificity since the model correctly labeled other classes as either COVID-19 or Normal, without mistakenly classifying them as Pneumonia. Experiment 4 attained the highest overall Specificity across the five experiments, with a value of 99.31%. This is attributed to the experiment's superior Recall values for the COVID-19 and Pneumonia classes. When considering the best Specificity value for each class across the experiments, we observe that experiment 2 achieved the highest Specificity for the Pneumonia class, with a value of 99.68%. This outcome can be attributed to the model's excellent COVID-19 Recall across the five experiments and its comparatively high Recall for the Normal class. Experiment 2 had the lowest number of FPs for the Pneumonia class, with a value of six, whereas experiment 1, experiment 3, experiment 4, and experiment 5 had 10, 14, 8, and 16 FPs, respectively. The model correctly identified the patients who did not have Pneumonia in experiment 2.

Experiment 3 demonstrates the highest Specificity for COVID-19, which can be attributed to its superior Recall for the Normal class across the five experiments and a comparably high Recall for Pneumonia. Examining the number of False Positives (FPs) for the COVID-19 class in experiment 3 (as shown in Fig. 19), we observe a value of 9, whereas experiment 1 has 16 FPs, experiment 2 has 15 FPs, experiment 4 has 19 FPs, and experiment 5 has 10 FPs.

**Figure 22:**
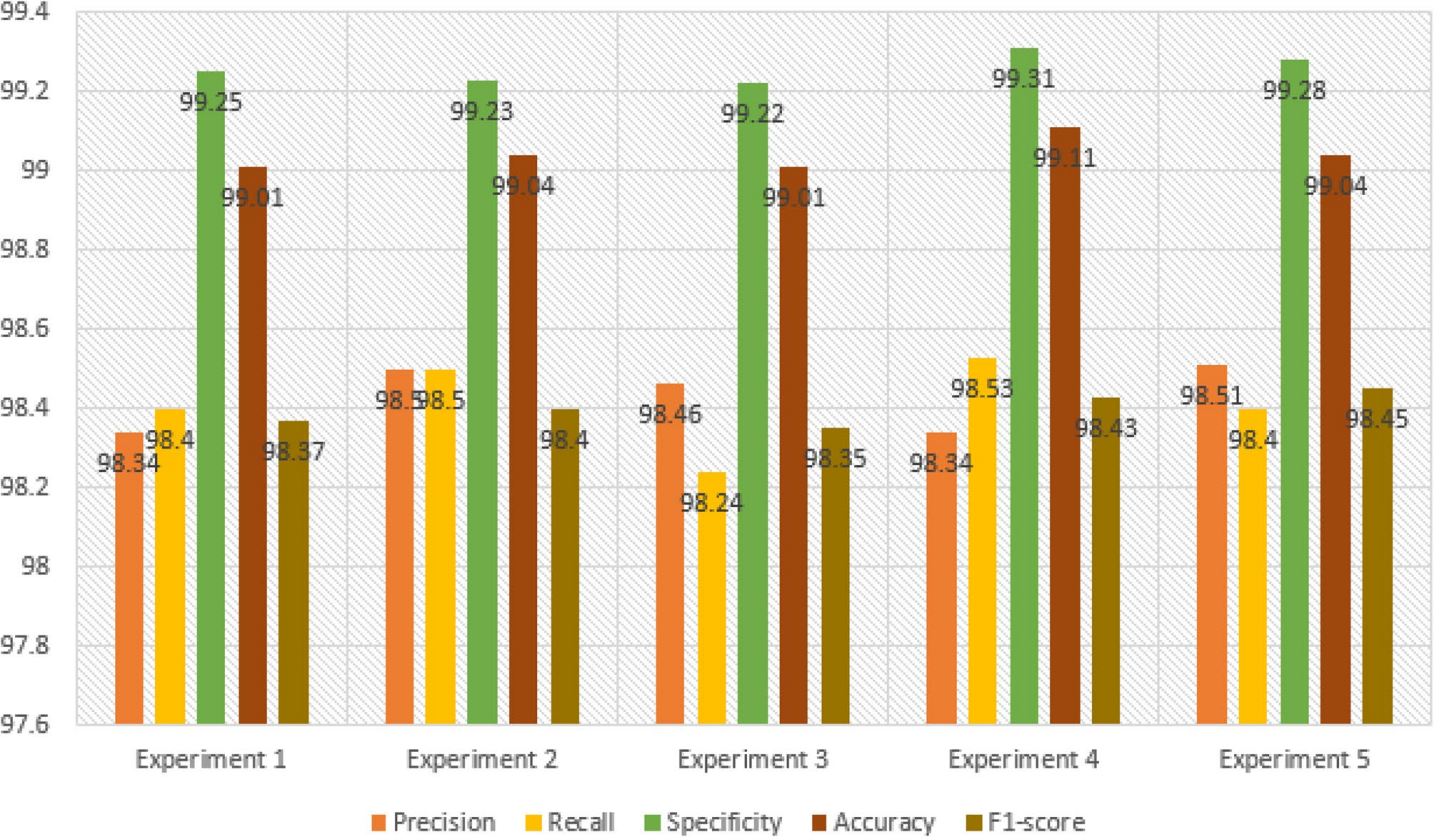
CovNetBoost performance metrics across the five experiments.

On the other hand, experiment 4 achieves the best Specificity for the Normal class, with a value of 99.33%. This high Specificity can be attributed to experiment 4's superior COVID-19 Recall score across the five experiments and its notably high Pneumonia Recall value, which ranks among the best. Analyzing the number of FPs that influence the Specificity score, we find that experiment 4 has a low number of FPs, specifically 9. In comparison, experiment 1 has 12 FPs, experiment 2 has 16 FPs, experiment 3 has 15 FPs, and experiment 5 has 15 FPs. Figure 22 presents a comprehensive overview of all the performance metrics obtained in each of the five experiments conducted for the CovNetBoost model.

### Comparison with state-of-the-art models on CovidxCT-2A dataset

In this section, we conduct a comparison between our models and other state-of-the-art models developed in the study^24^ based on Precision, Recall, Specificity, Accuracy, and F1-score. Table 15 presents the performance metrics for testing on the CovidxCT-2A dataset, with our models listed at the end of the table.

**Table 15:** Performance metrics obtained for our models, including other models in the literature when testing on the CovidxCT-2A dataset.

Net	Precision	Recall	Specificity	Accuracy	F1-score
AlexNet	91.88%	92.50%	96.64%	95.38%	92.17%
GoogLeNet	89.46%	88.96%	95.13%	93.59%	89.09%
InceptionV3	95.48%	94.34%	97.54%	96.97%	94.84%
VGG-16	96.65%	96.57%	98.44%	97.93%	96.58%
VGG-19	97.85%	97.87%	99.08%	98.87%	97.86%
ShuffleNet	95.36%	94.92%	97.94%	97.28%	95.13%
MobileNetV2	94.24%	93.05%	97.31%	96.38%	93.38%
ResNet-18	96.41%	96.67%	98.22%	97.71%	95.98%
ResNet-50	92.19%	89.53%	95.61%	94.62%	90.45%
ResNet-101	94.99%	93.06%	97.16%	96.53%	93.08%
CovNetBoost (ours)	98.43%	98.41%	99.26%	99.04%	98.42%
Stand-alone CNN (ours)	98.25%	98.44%	99.27%	98.97%	98.34%

**Figure 23:**
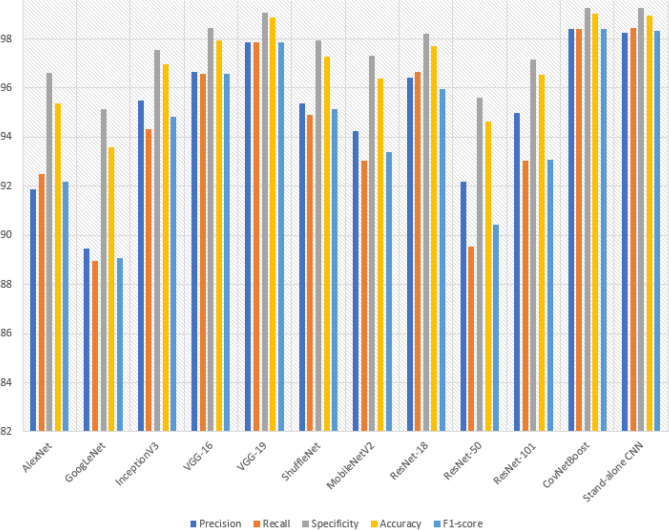
Performance metrics for all models.

As depicted in Fig. 23, our two models, CovNetBoost and the stand-alone CNN, have outperformed all the models proposed in the study^24^. We have successfully surpassed all 10 models introduced by the authors in^24^. The exceptional performance of our models can be attributed to hyper-parameter optimization for both the CNN and XGBoost models, as well as the incorporation of Transfer Learning, Batch Normalization, and Dropout techniques. These mechanisms have enabled our models to generalize effectively on unseen data and mitigate the risk of overfitting. Among the two models we proposed, the hybrid CNN-XGBoost model achieved the highest classification accuracy of 99.04%, surpassing the performance of the stand-alone CNN, which attained an accuracy of 98.97%. These results fulfill objectives 3 and 4 of our study. The combination of CNN and XGBoost has demonstrated promising outcomes and warrants further investigation to enhance this hybrid model.

In the study^24^, it was mentioned that the VGG-19 architecture performed exceptionally well, with an overall classification accuracy of 98.87%. The results obtained from the VGG-19 model were encouraging, and exploring this architecture further would be worthwhile. Additionally, the authors of^24^ compared their best-performing model, the VGG-19 architecture, with other existing COVID-19 classification models. Similarly, in Table 16, we have benchmarked the results of our two models, CovNetBoost and the stand-alone CNN, against the accuracy results of state-of-the-art models from the literature^24^.

**Table 16:** Classification accuracy obtained when testing on the CovidxCT-2A dataset.

Net	Accuracy
COVID-Net CT-1	94.5%
COVID-Net CT-2L	98.1%
COVID-Net CT-2 S	97.9%
Bit-M	99.2%
VGG-19	98.87%
CovNetBoost (this work)	99.04%
Stand-alone CNN (this work)	98.97%

In Table 16, we observe that the Bit-M model achieved the highest classification accuracy of 99.2%, followed by our two models, CovNetBoost and the stand-alone CNN, with accuracies of 99.04% and 98.97%, respectively. The VGG-19 model, which was identified as their best performing model^24^, obtained a classification accuracy of 98.87% and ranks 4th in the table. While our primary focus was to compare our two models with the proposed COVID-19 classification models in^24^, we will briefly discuss the results presented in Table 16 and explore the reasons behind our models falling short of outperforming the Bit-M model.

The authors of the Bit-M model provided crucial details that shed light on why our models did not surpass their classification accuracy. Specifically, in terms of the COVID-19 class, they achieved Recall, Precision, and Specificity values of 98.7%, 98.5%, and 99.5%, respectively^7^. In comparison, our hybrid model achieved Recall, Precision, and Specificity values of 97.16%, 97.72%, and 99.25%, respectively. Similarly, the stand-alone CNN achieved Recall, Precision, and Specificity values of 97.49%, 97.82%, and 99.34%, respectively. It is evident that the Bit-M model performed significantly better than our two models in terms of COVID-19 classification, as indicated by these metrics from^7^. While our models were unable to surpass the classification score of the Bit-M model, it is important to analyze the specific details and techniques employed in the Bit-M model that contributed to its superior performance.

## Conclusion

An ablation study investigates the performance of an AI system by removing certain components to understand the contribution of the component to the overall system In this study, our objective was to contribute to the research and understanding of COVID-19 by developing two models: CovNetBoost, a hybrid model, and a classical stand-alone CNN. These models were designed to accurately classify patients with COVID-19, Pneumonia, and Normal patients (without any disease). The hybrid model, CovNetBoost, combines the use of a CNN as a feature extractor and XGBoost, a high-performing classifier. On the other hand, the stand-alone CNN employs Dense layers for classification. To improve the performance of both models, we optimized their hyperparameters using Bayesian optimization. This allowed us to "cheat-start" the training process with optimal settings. Additionally, we incorporated techniques such as Transfer learning, Dropout, and Batch normalization to prevent overfitting of the models.

To evaluate the effectiveness of our models, we conducted a benchmarking analysis against other state-of-the-art models in the literature. Our proposed models outperformed all the models from a previous study in terms of Precision, Recall, Specificity, Accuracy, and F1-score. Specifically, the hybrid model achieved impressive results with Precision of 98.43%, Recall of 98.41%, Specificity of 99.26%, Accuracy of 99.04%, and F1-score of 98.42%. Similarly, the stand-alone CNN model exhibited high performance, achieving Precision of 98.25%, Recall of 98.44%, Specificity of 99.27%, Accuracy of 98.97%, and F1-score of 98.34%.

The success of our proposed models can be attributed to the optimization of hyperparameters for both the CNN and XGBoost, as well as the implementation of Transfer learning, Batch Normalization, and Dropout techniques. Notably, the combination of CNN and XGBoost yielded the best classification accuracy of 99.04%, surpassing the performance of the stand-alone CNN and all models in the previous study. Our hybrid model presents a promising avenue for further research and improvement to achieve applicability in real-life scenarios. However, it is essential to acknowledge the limitations of our study. One limitation is the issue of imbalanced data, which we attempted to address through resampling methods. Unfortunately, this led to the overemphasis of features from the under-represented classes, resulting in lower Recall and Precision values for COVID-19 compared to Pneumonia and Normal classes across multiple experiments.

Another limitation arises from the limited number of epochs used to train the feature extractors in both models. Due to power constraints during load shedding schedules, we could only train the models for a maximum of 10 epochs. Furthermore, we did not perform any pre-processing on the data, such as noise removal or image contrast adjustment for images with low contrast. Lastly, data augmentation techniques were not employed to increase the dataset size and introduce variations that could potentially enhance the model’s performance. Addressing these limitations in future studies will be crucial for further improving the performance and practicality of our models in real-world scenarios.

## Data Availability

The datasets used and/or analyzed during the current study available from the corresponding author on reasonable request.

## Abbreviations

CAP: Community acquired pneumonia
CT: Chest computed tomography
AUC: Area under the receiver characteristic curve
ROC: Receiver operating characteristic
PR: Precision recall
CNN: Convolutional neural network
CXR: Chest x-ray
VGG: Visual geometry group
BiLSTM: Bidirectional long short-term memories
ANN: Artificial neural network
MAC: Multiple accumulate operation
PPV: Pulse pressure variation
CAD: Computer aided diagnostic
ReLU: Rectified linear unit
ML: Machine learning
PEPX: Projection expansion projection extension
GBDT: Gradient boosted decision trees
GBM: Gradient boosting machine
TP: True positive
TN: True negative
FP: False positive
FN: False negative
